# Supplementary figures and images for: The pelvic organs receive no parasympathetic innervation
Source: eLife. 2024 Mar 15;12:RP91576. doi: 10.7554/eLife.91576 (PMC10942786; doi:10.7554/eLife.91576)

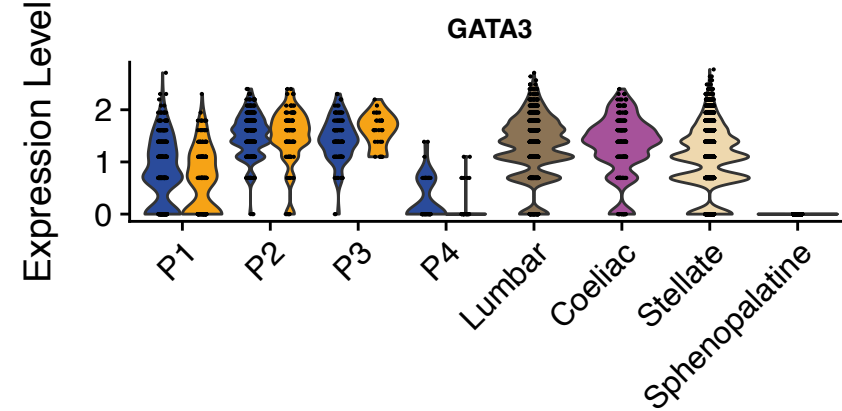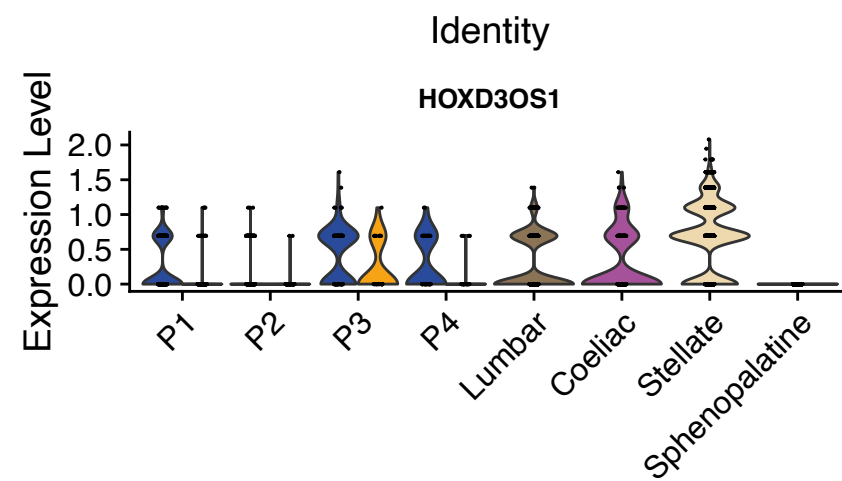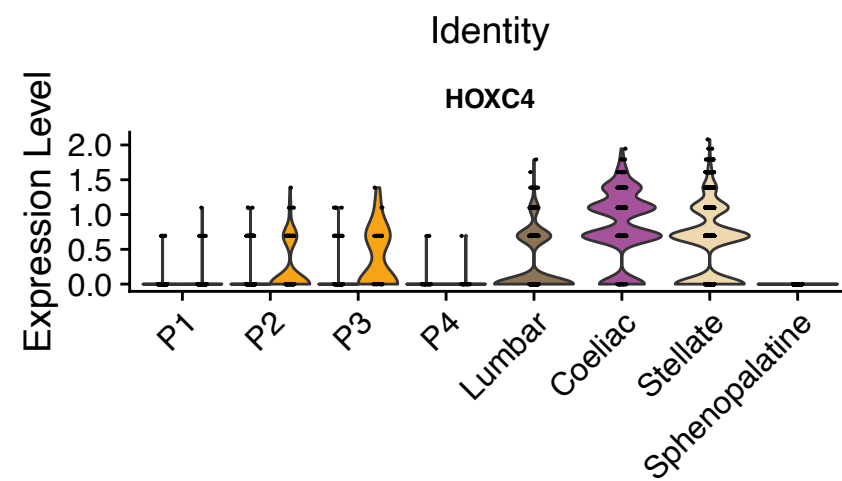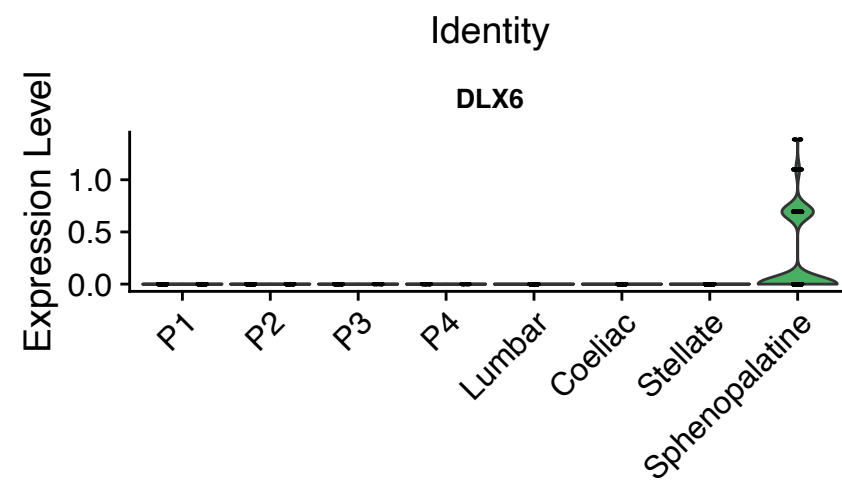

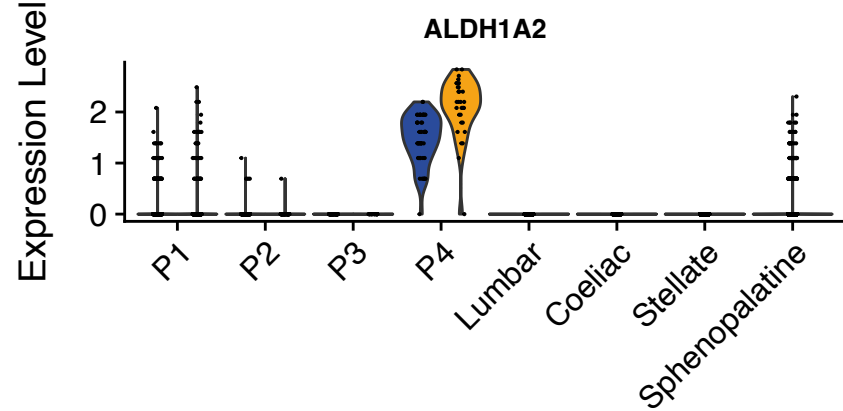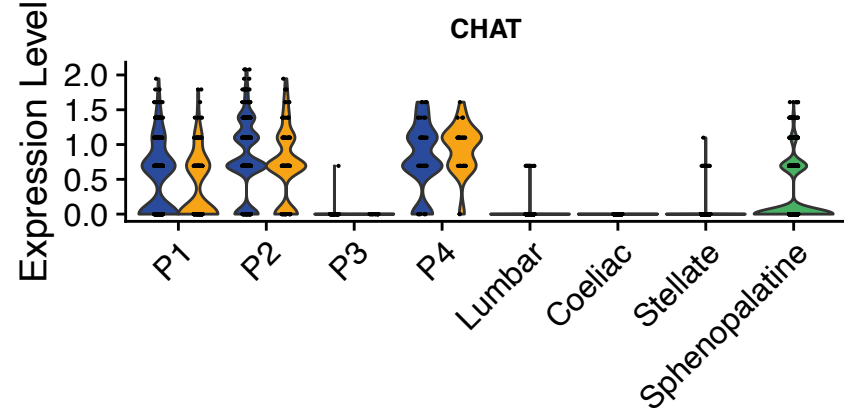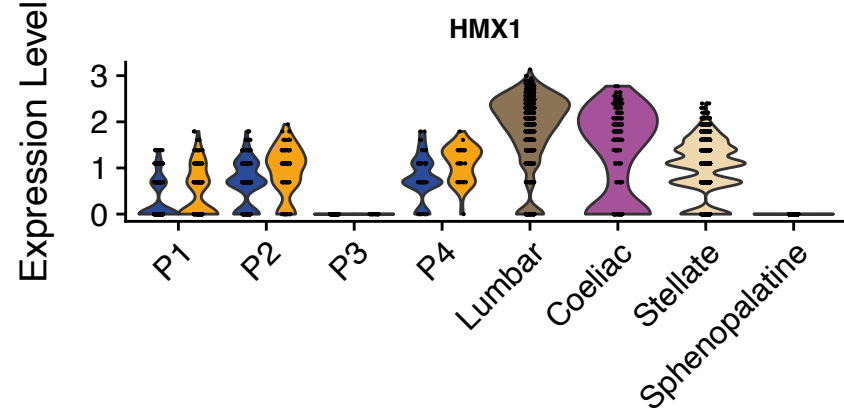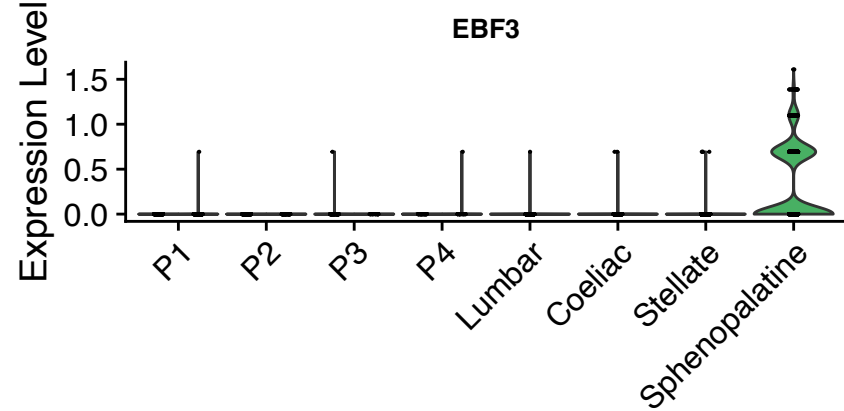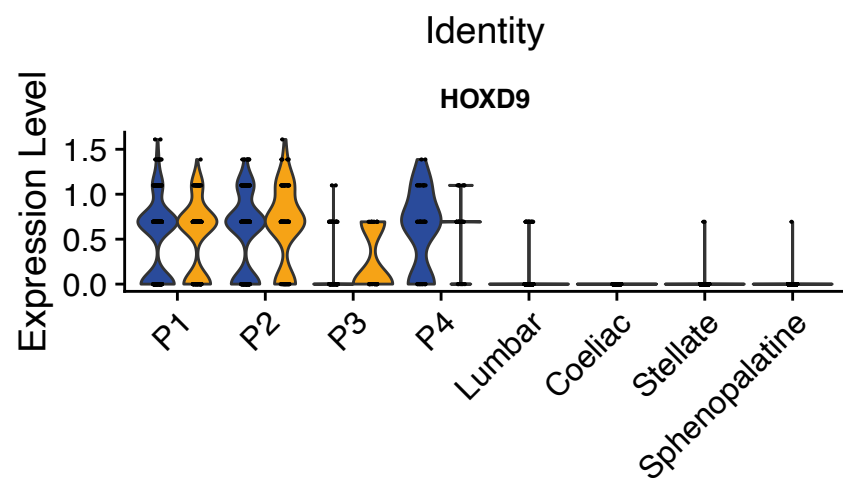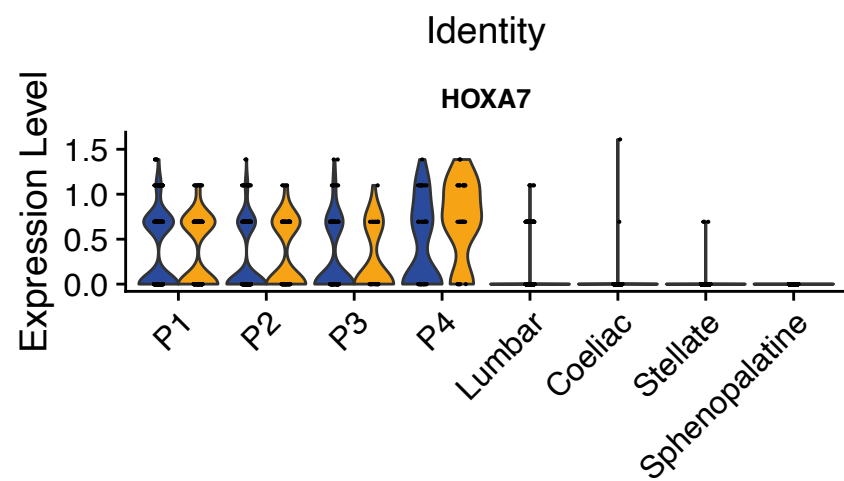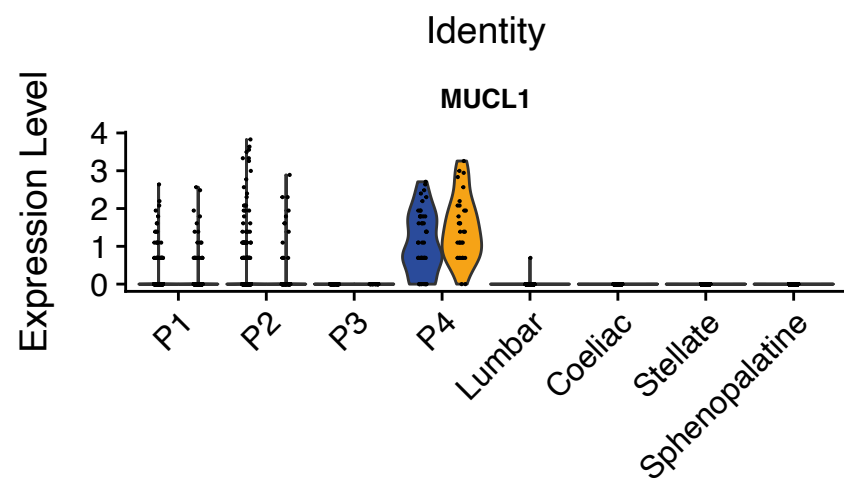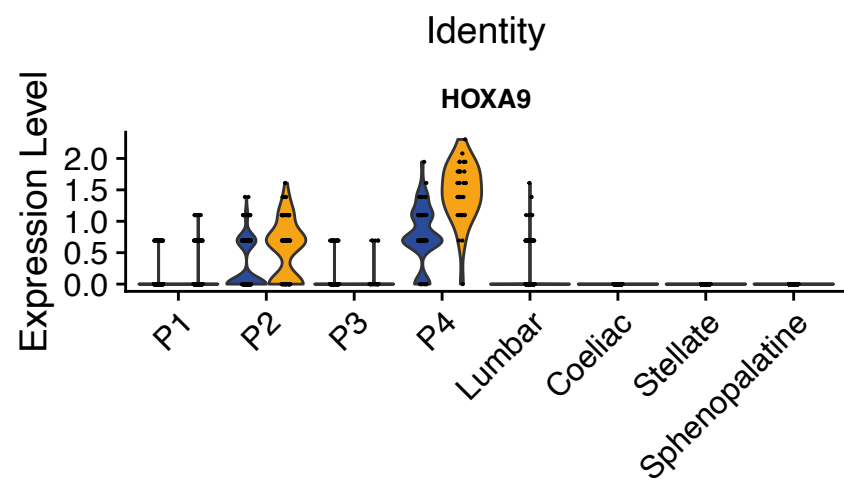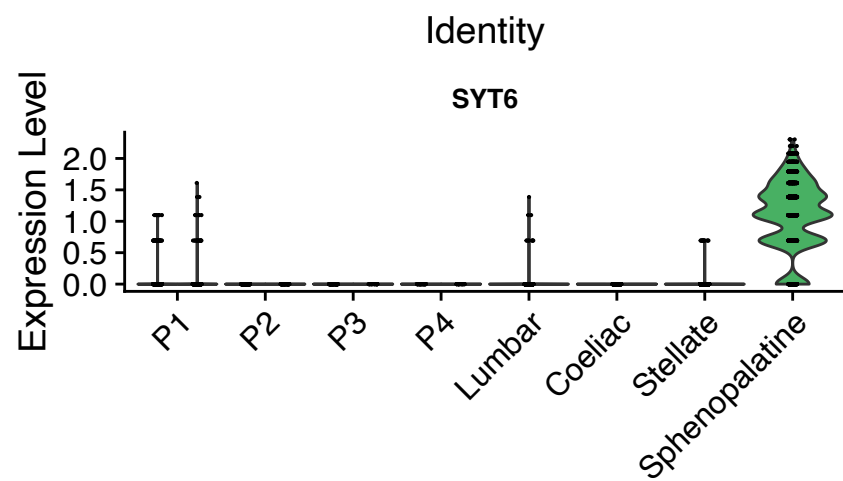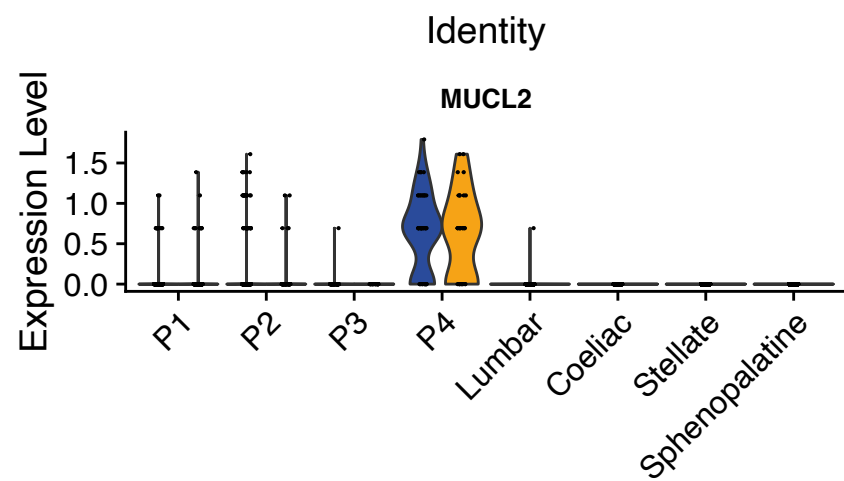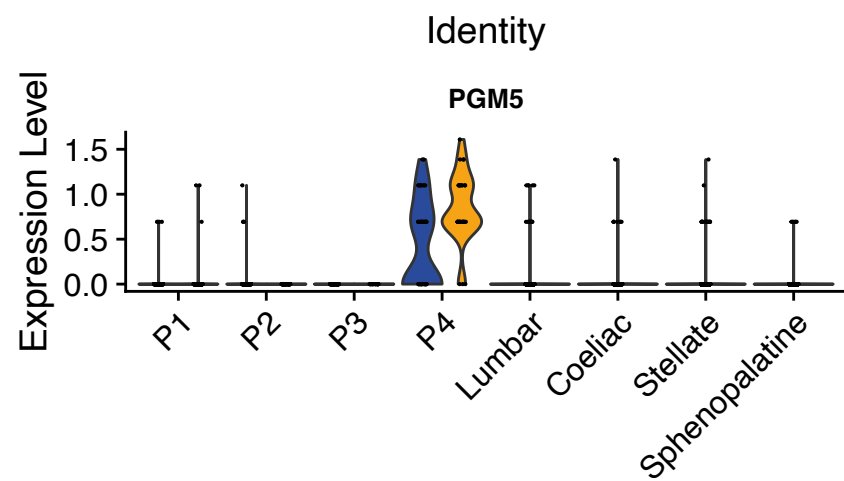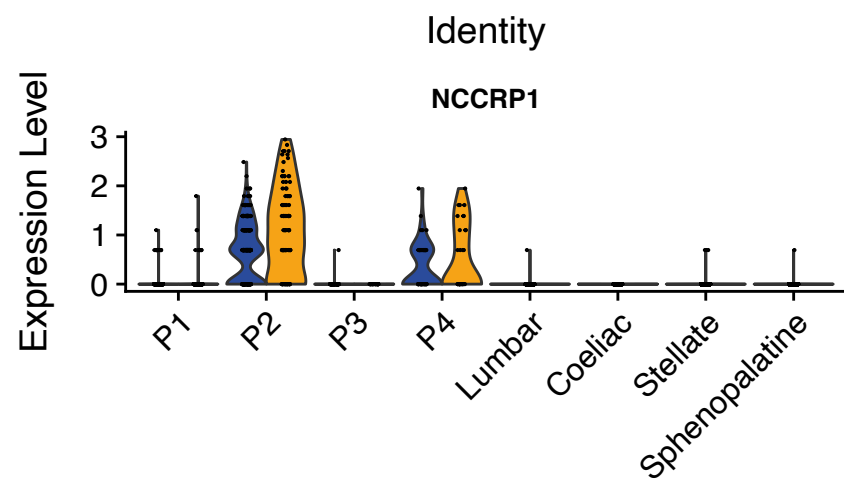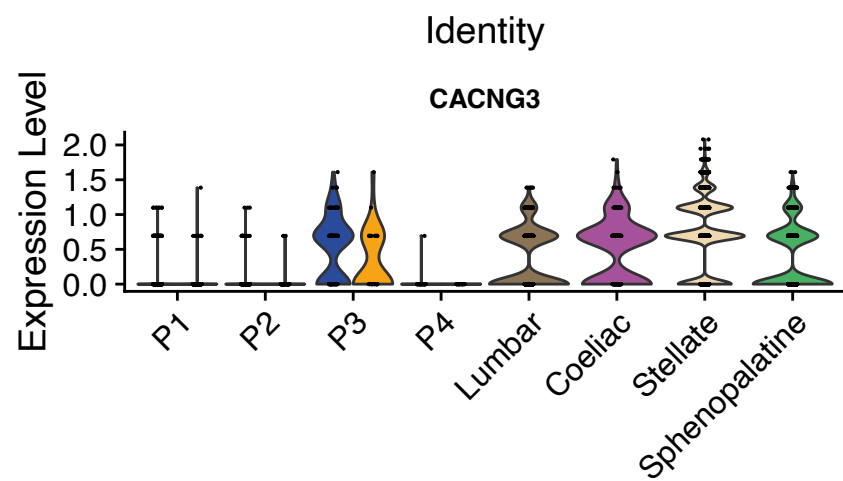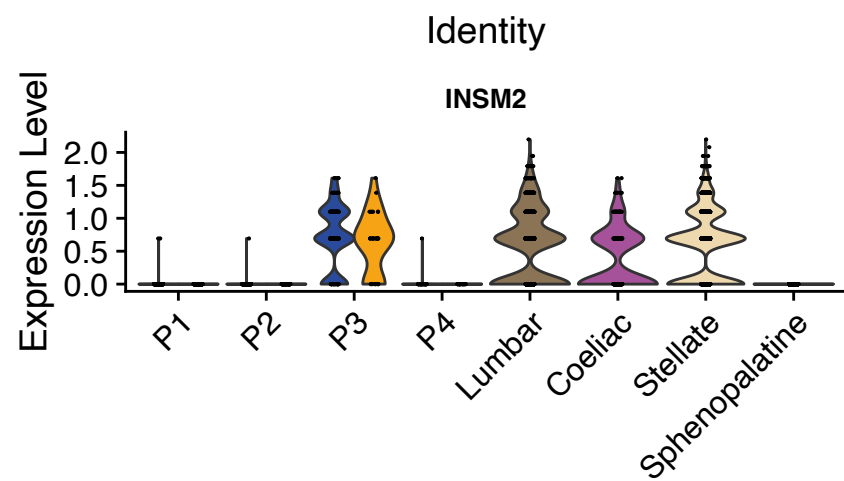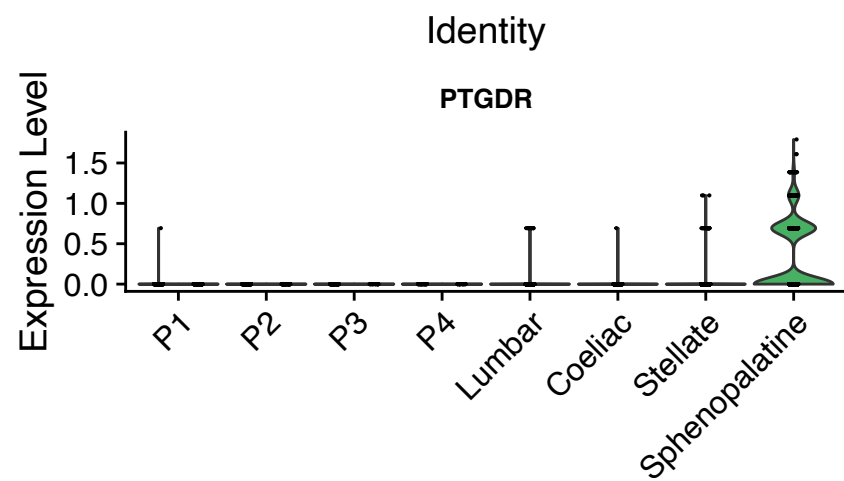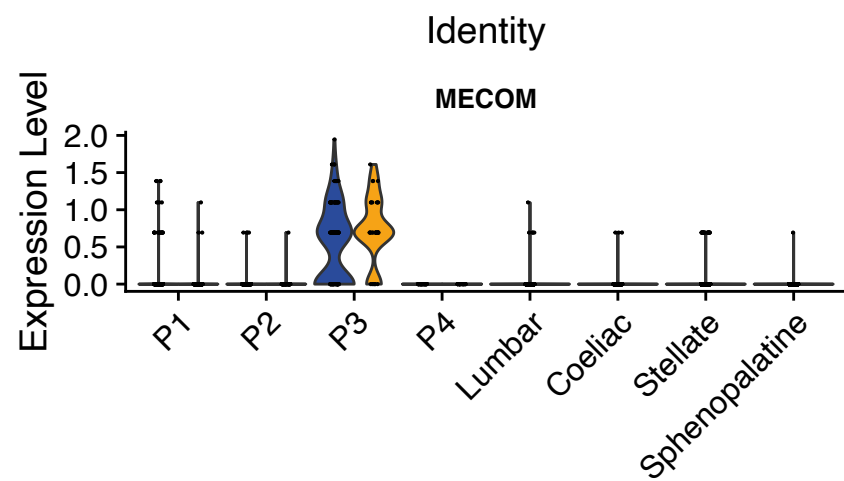

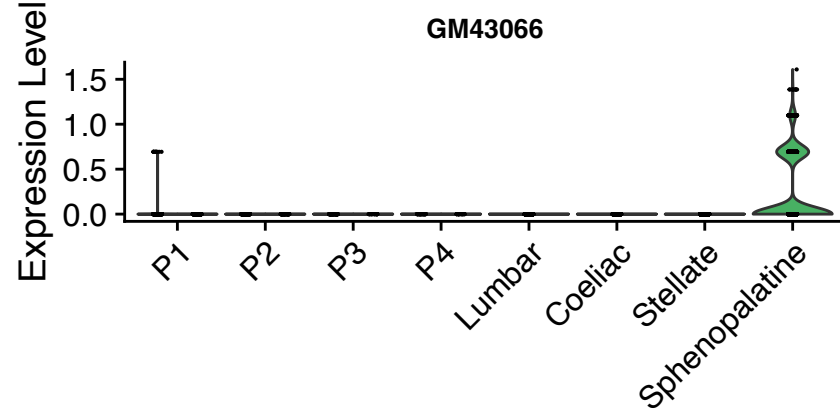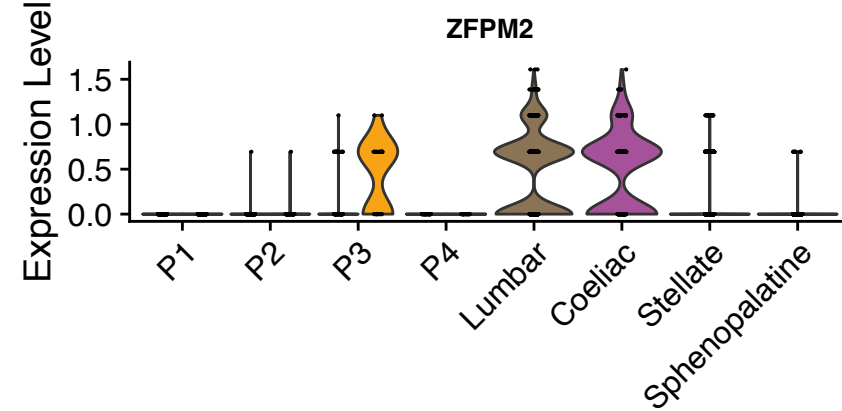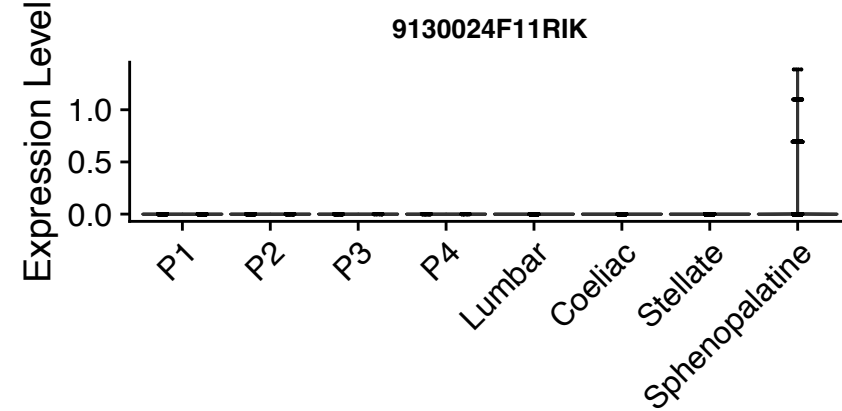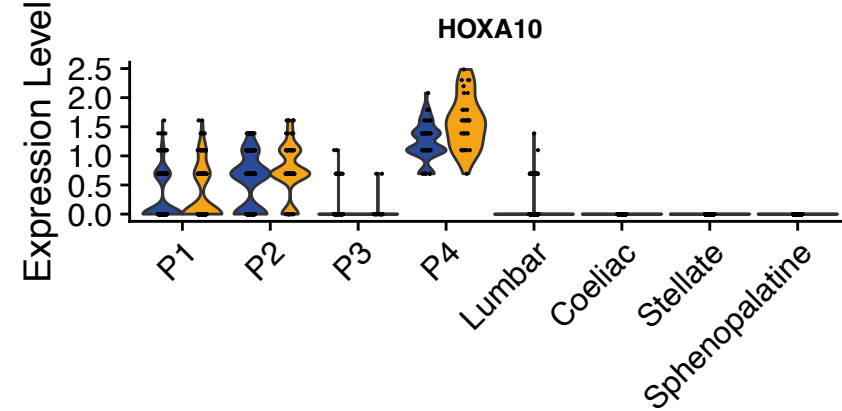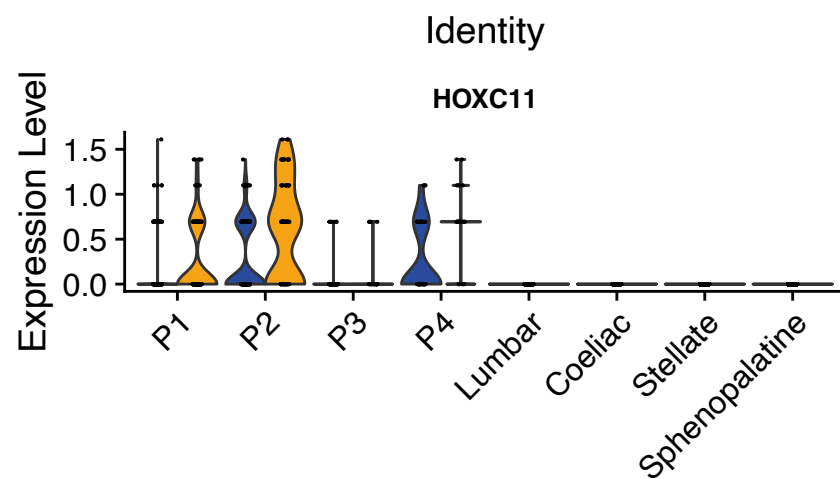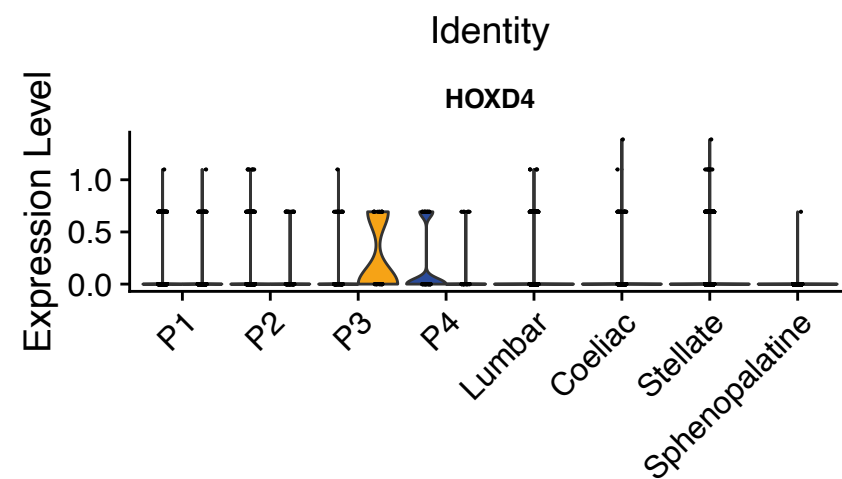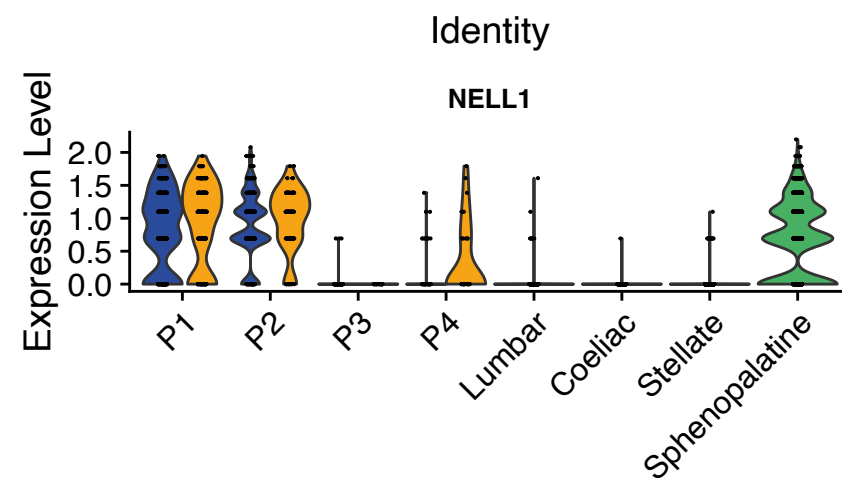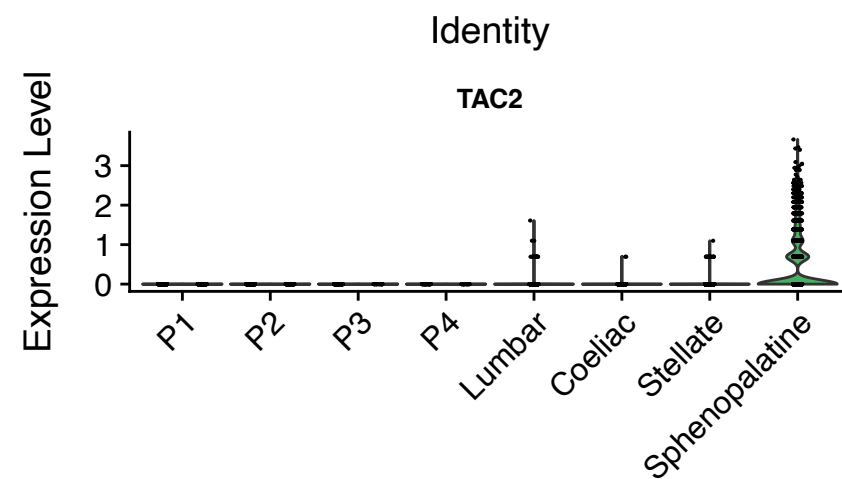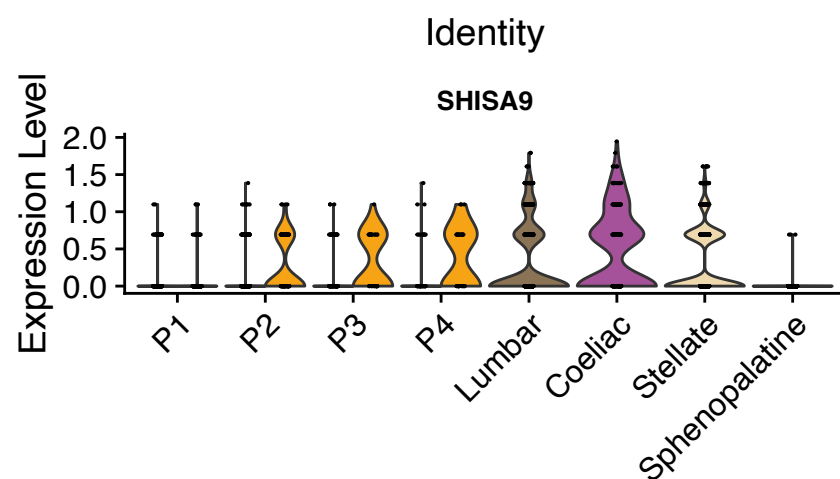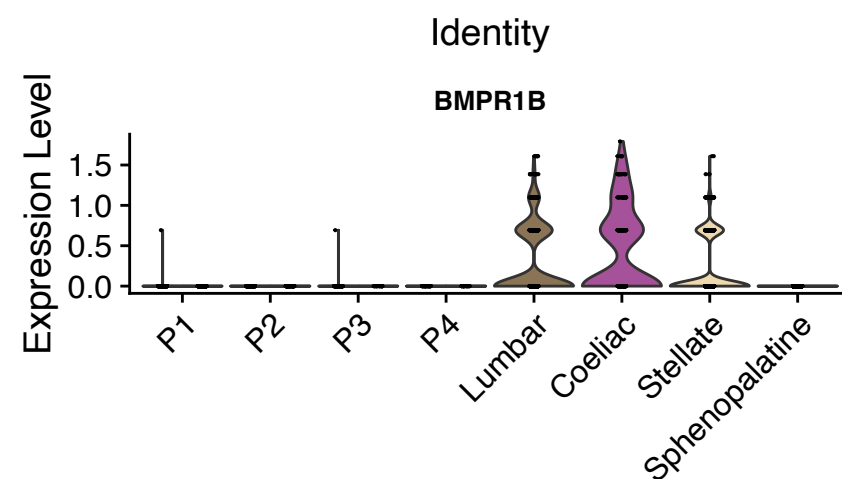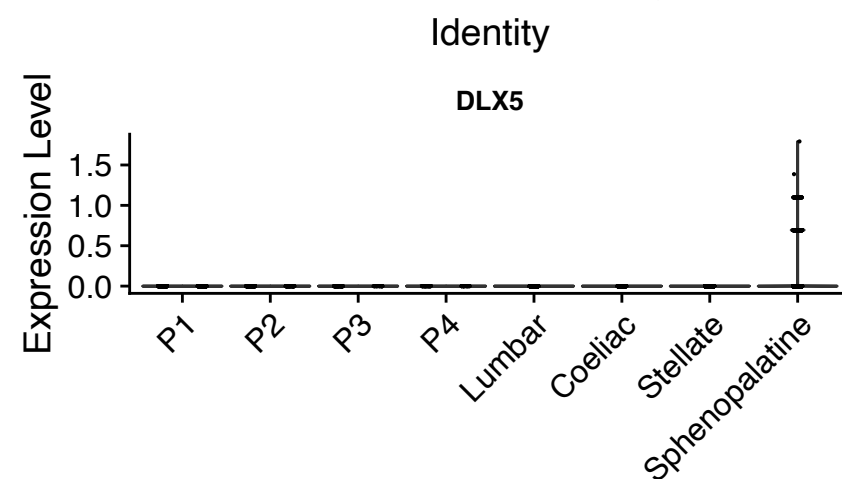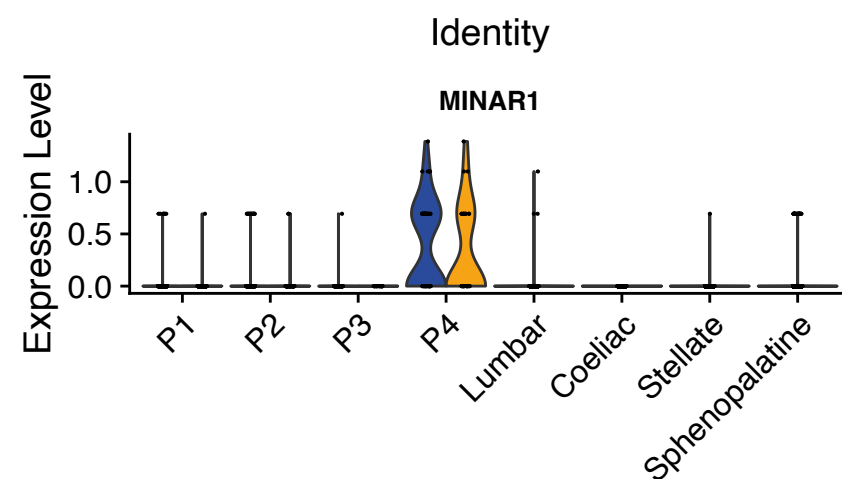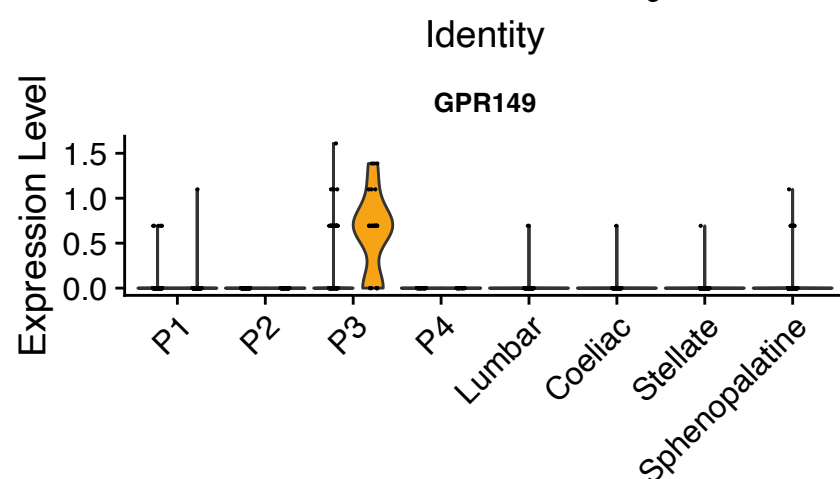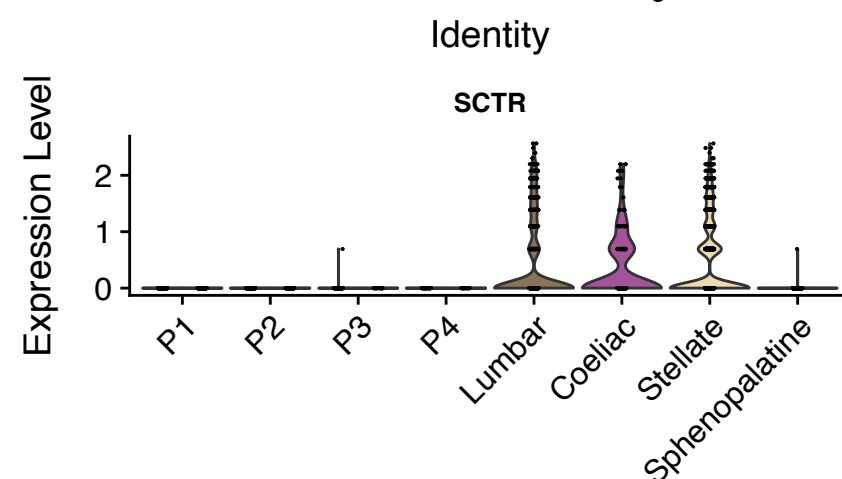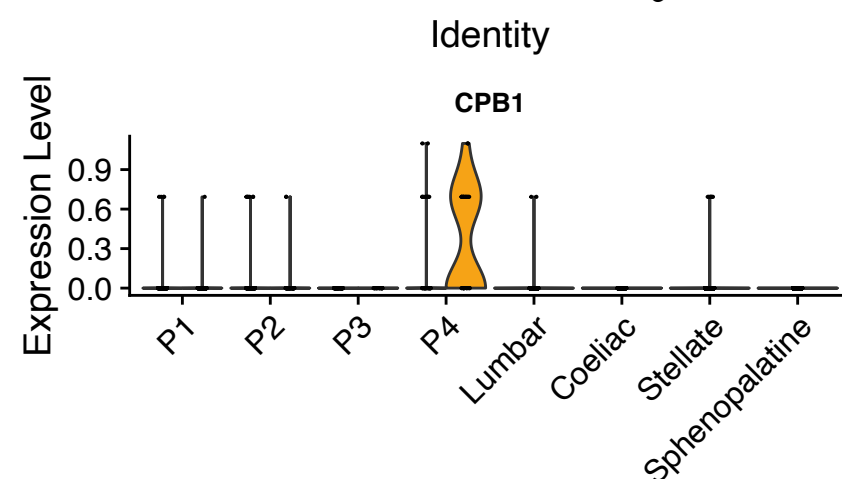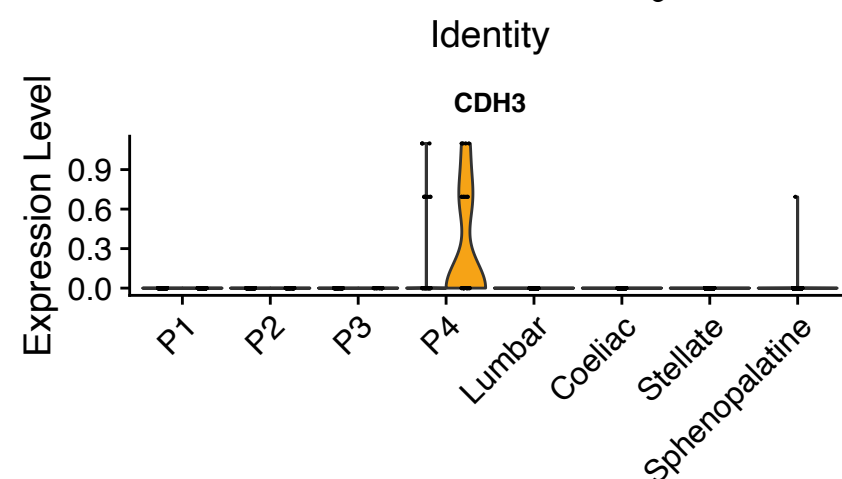

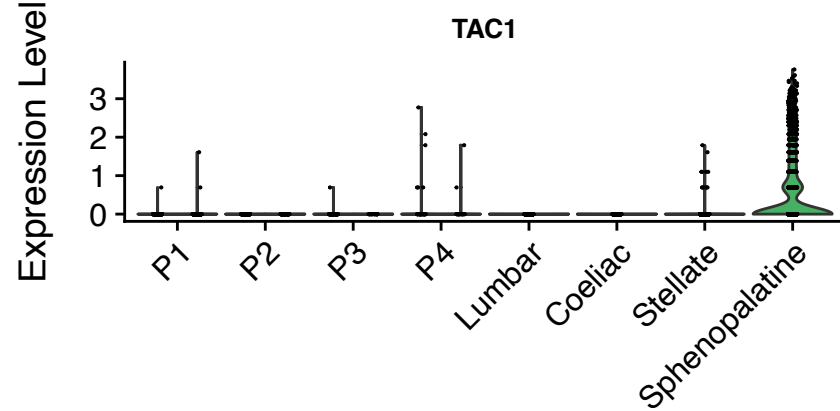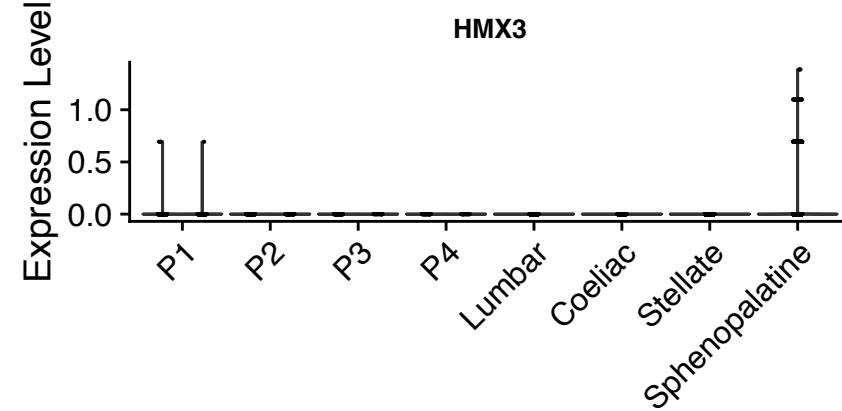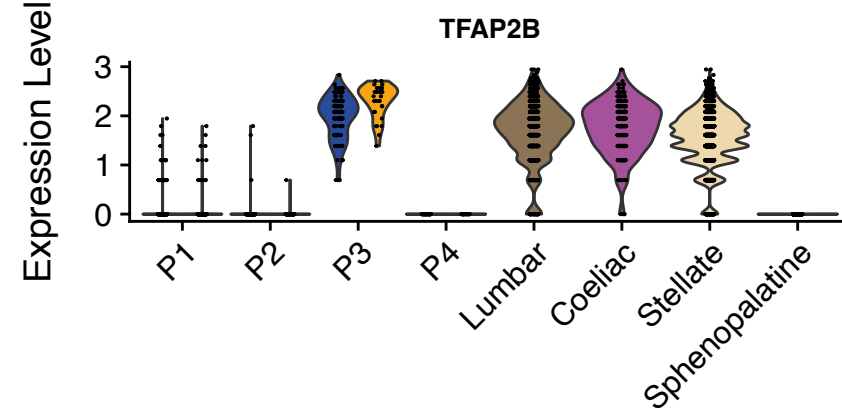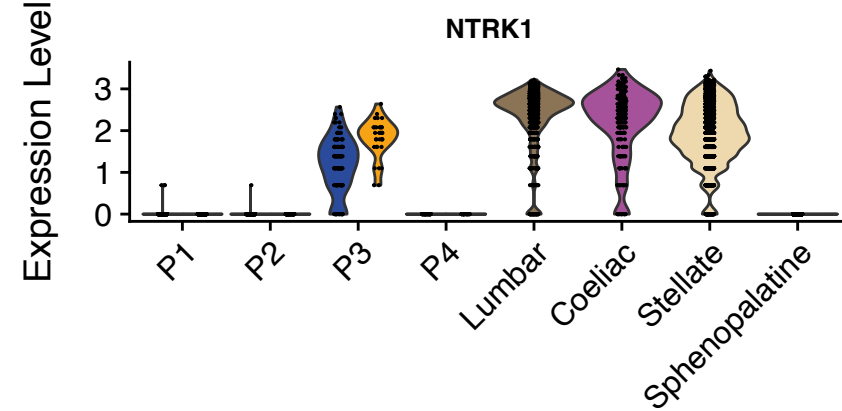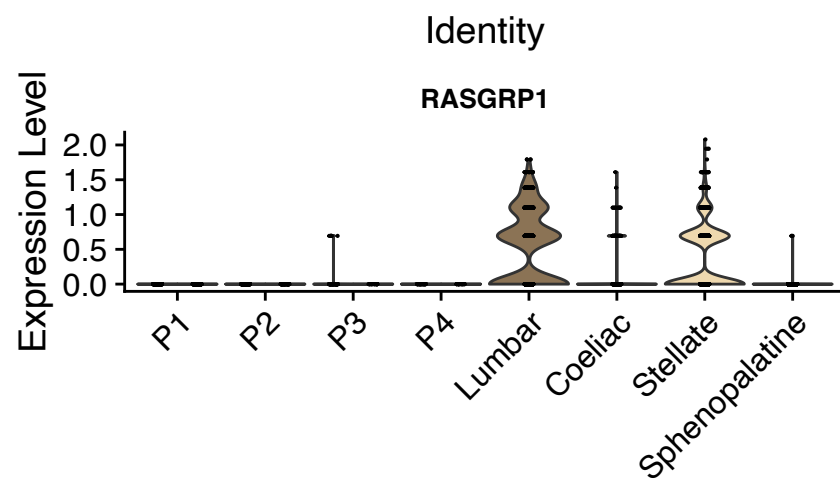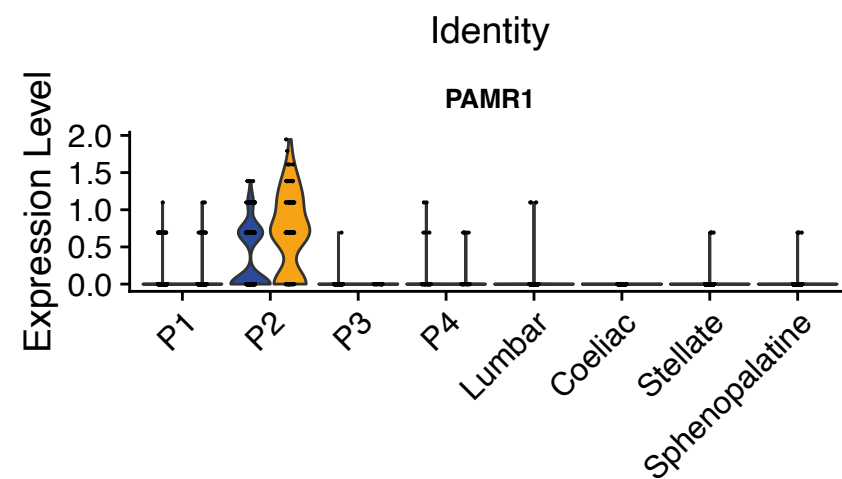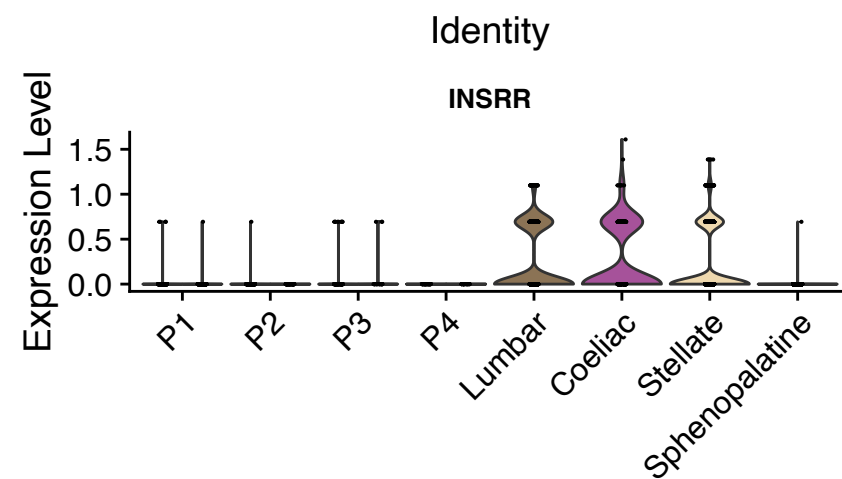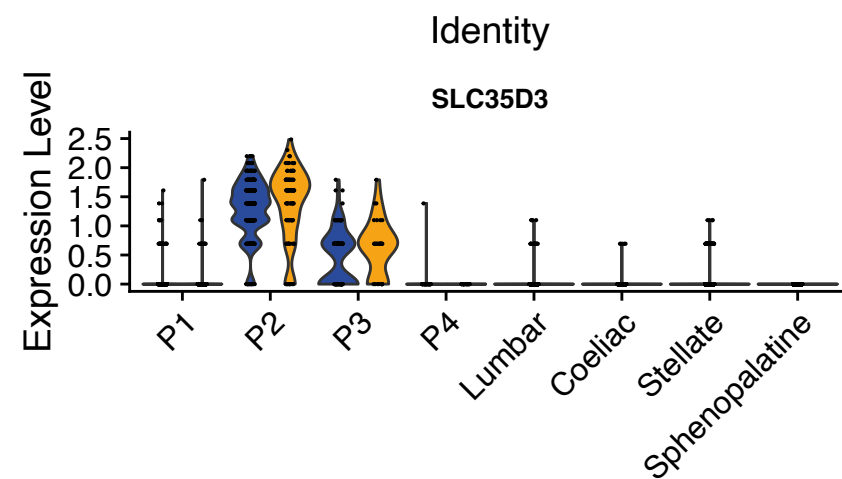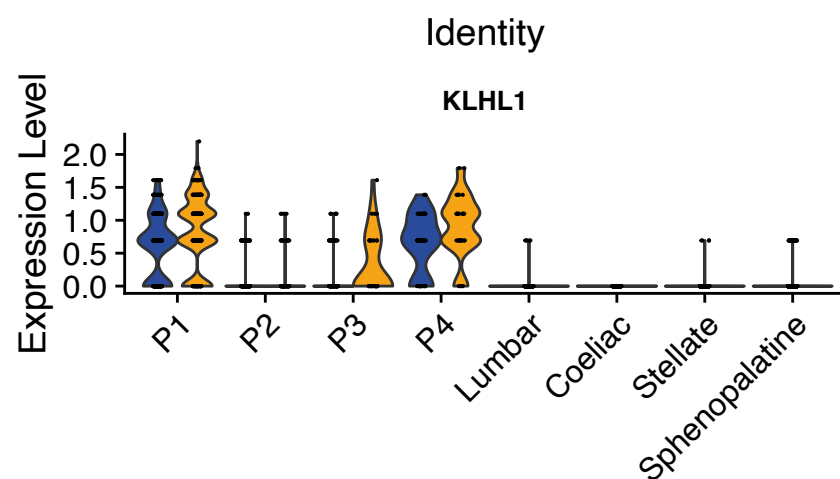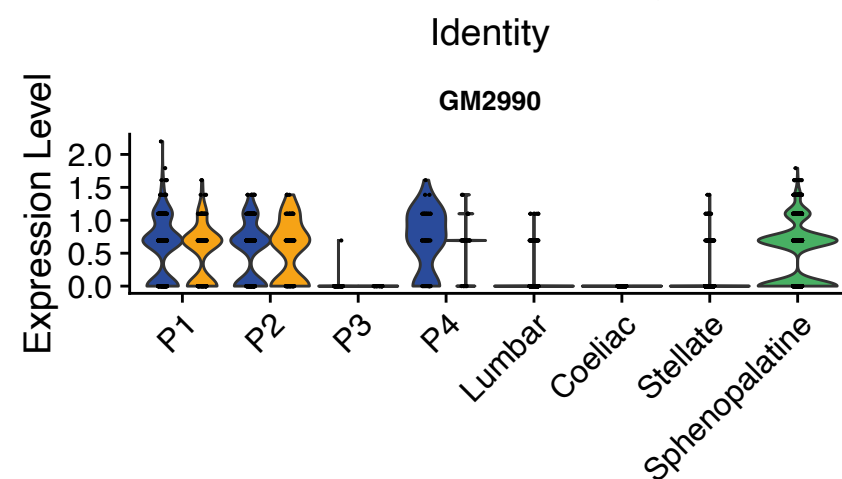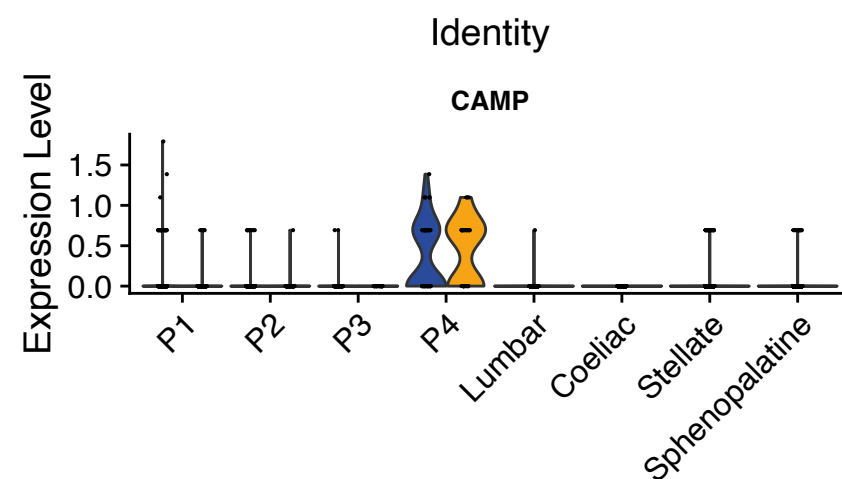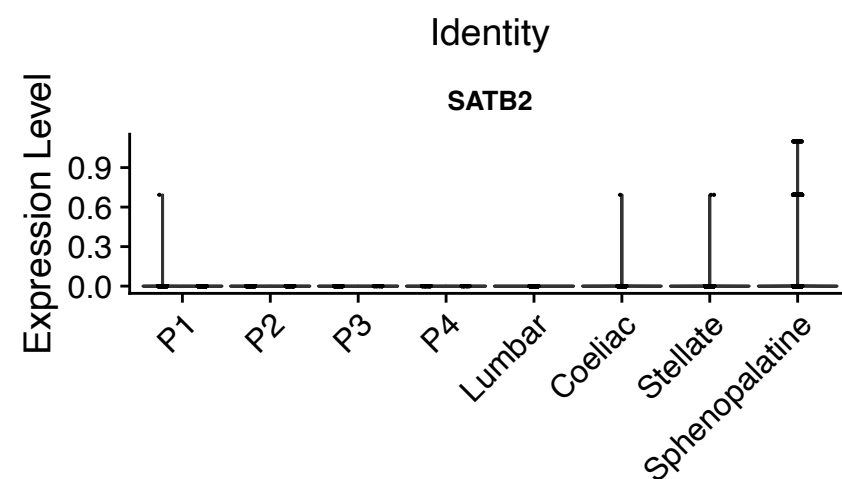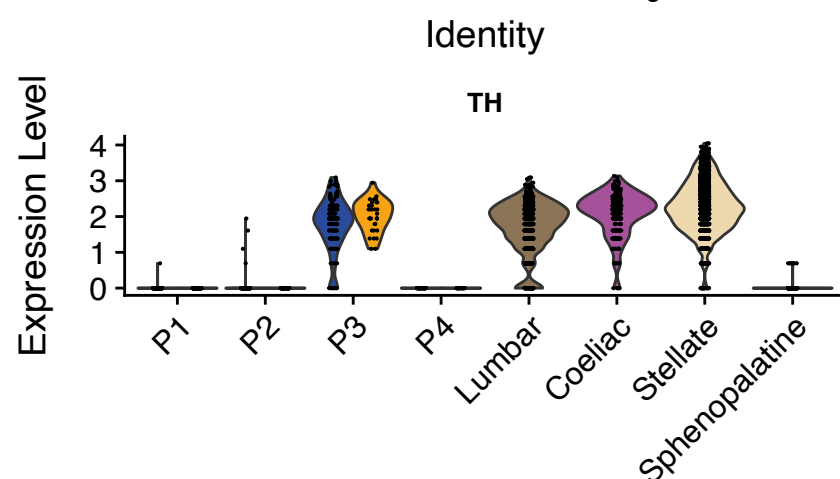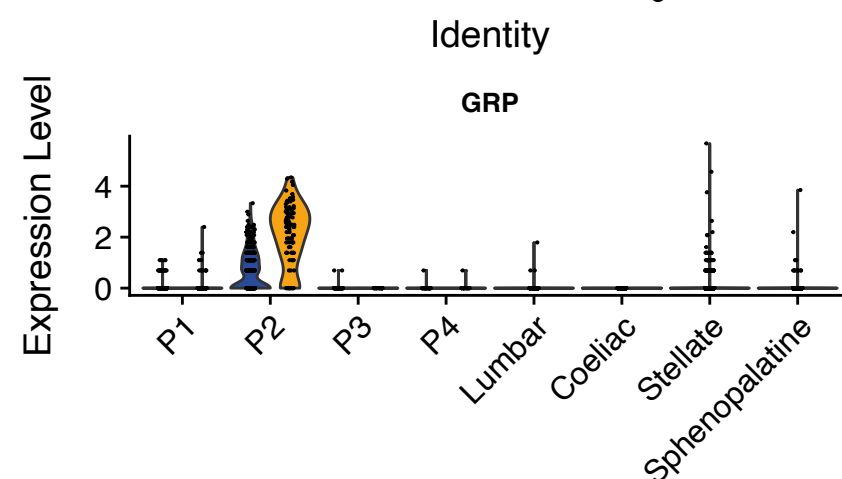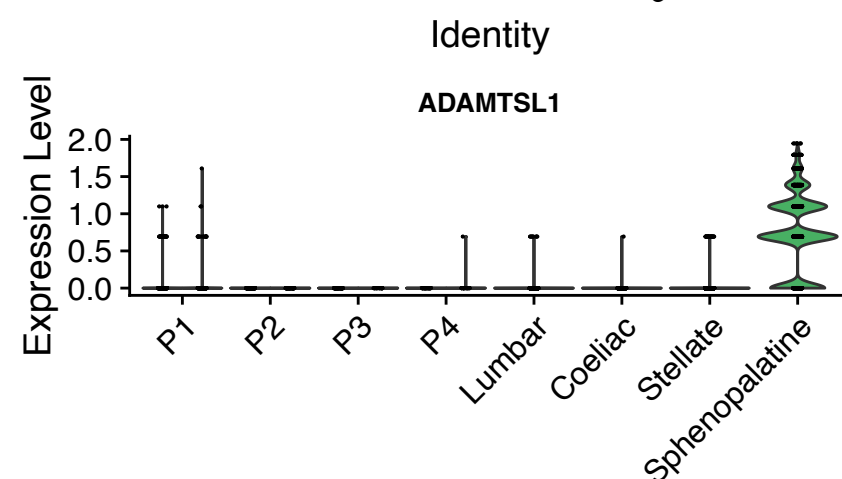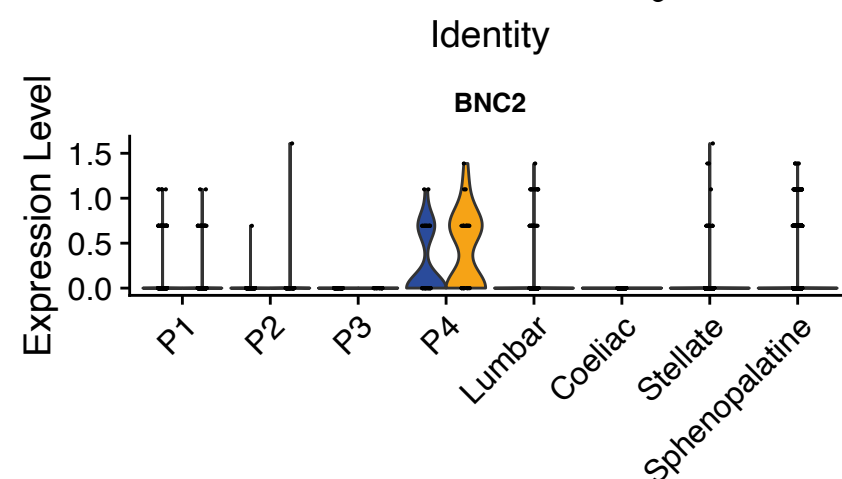

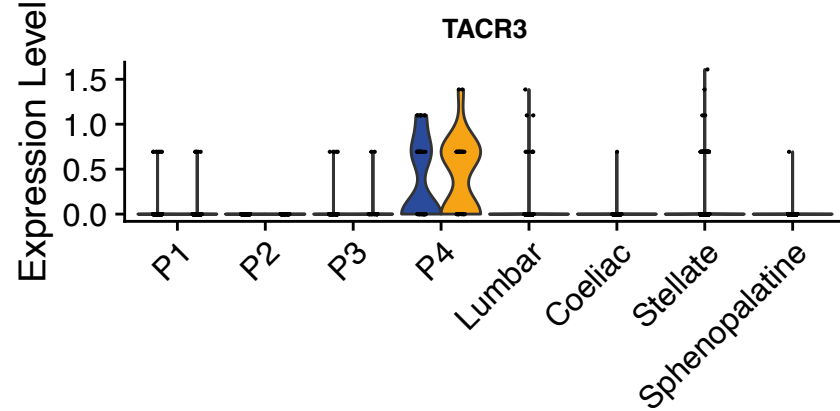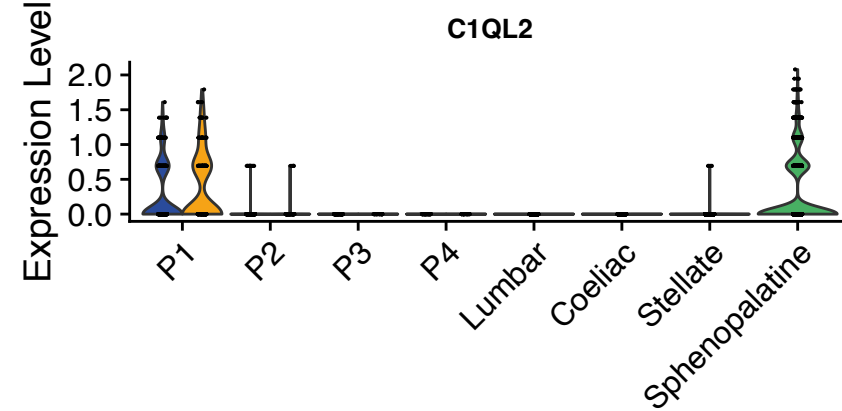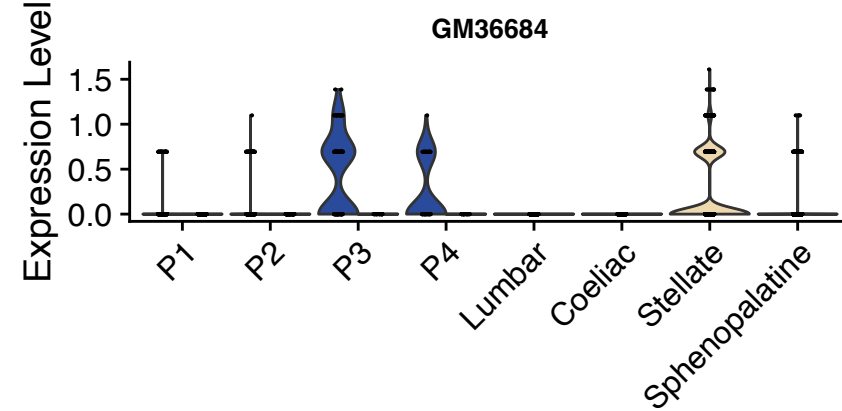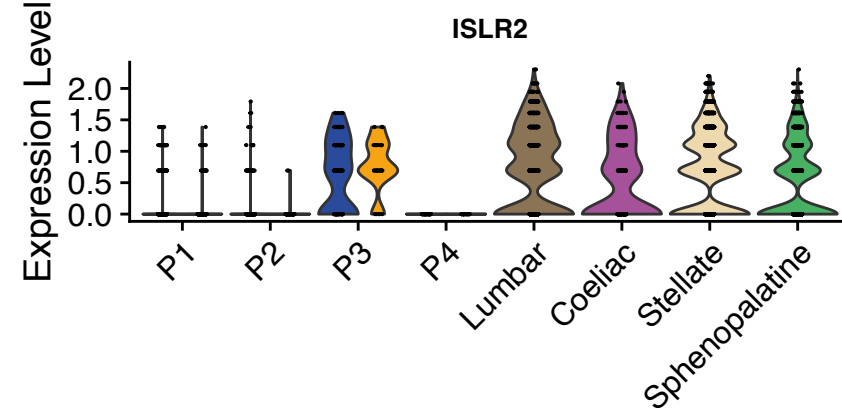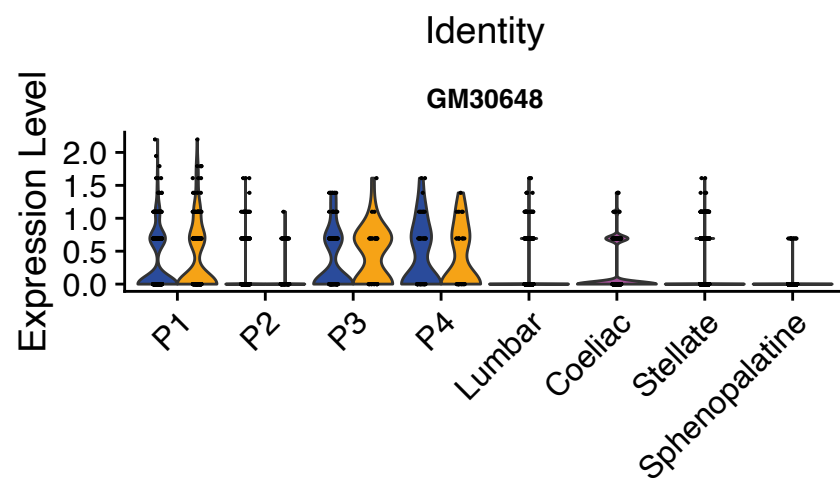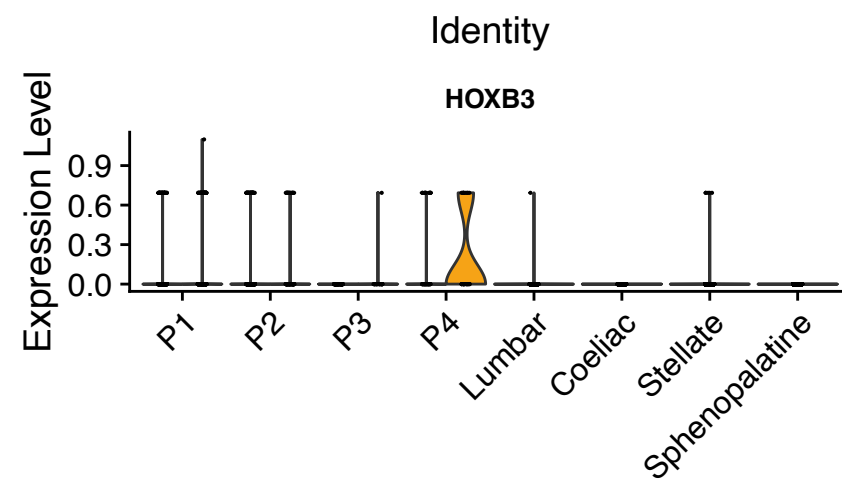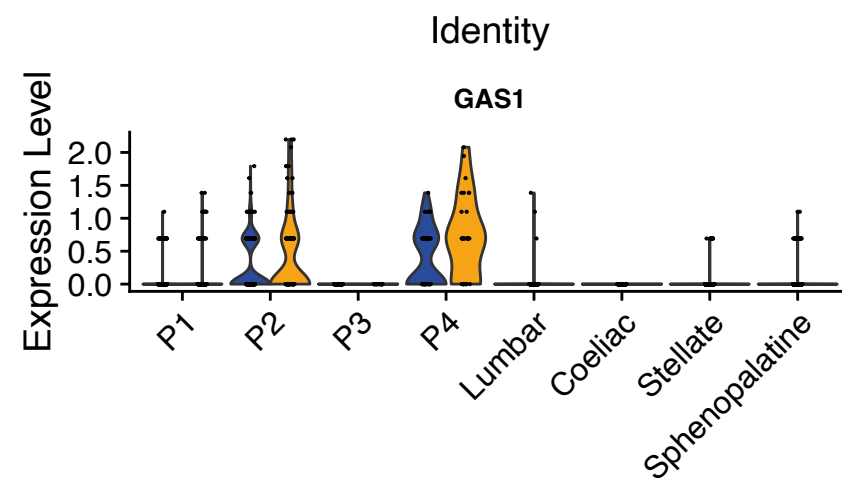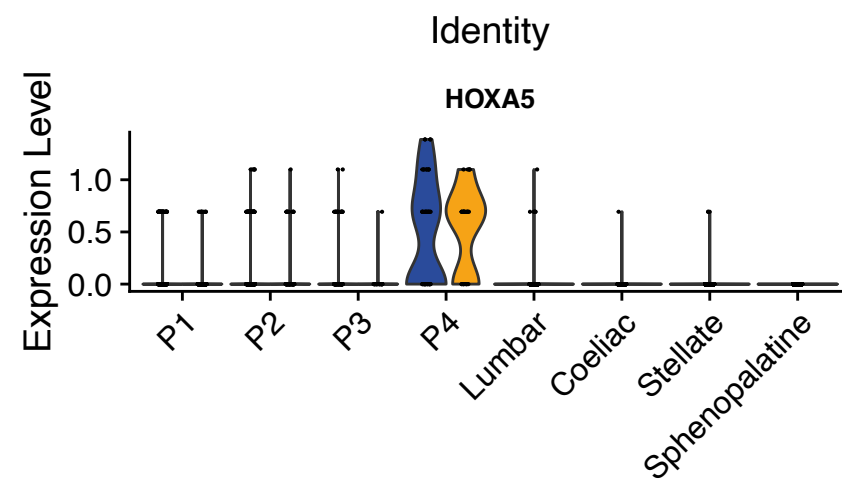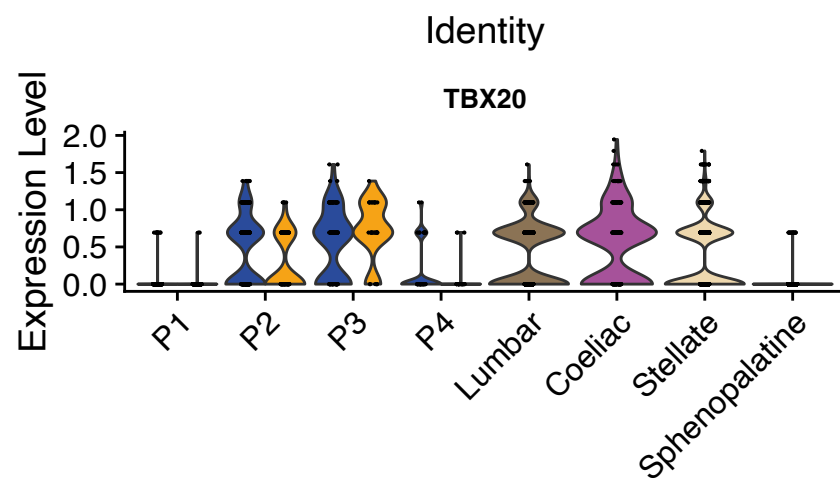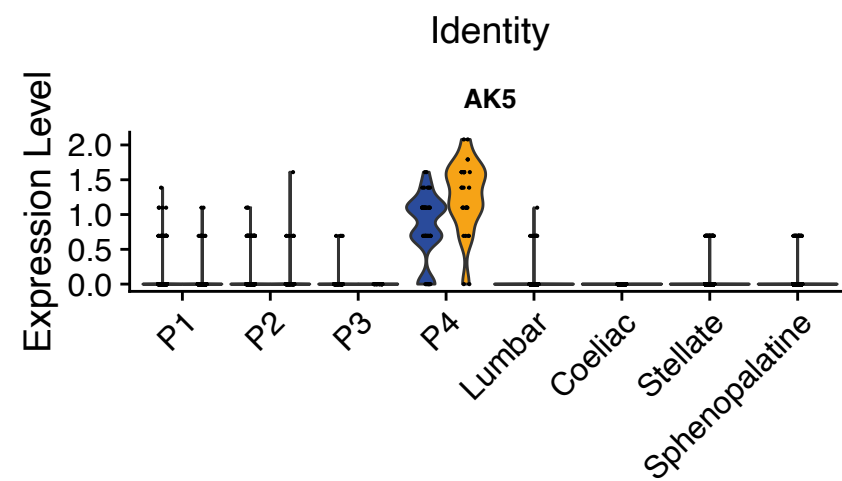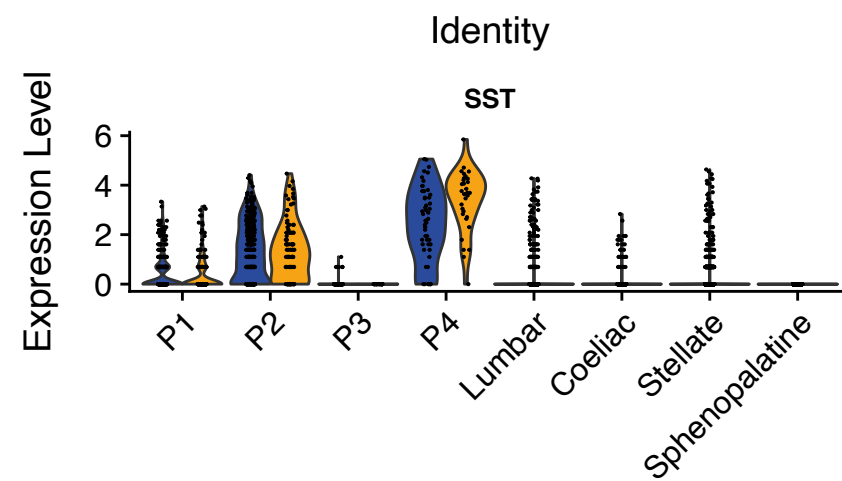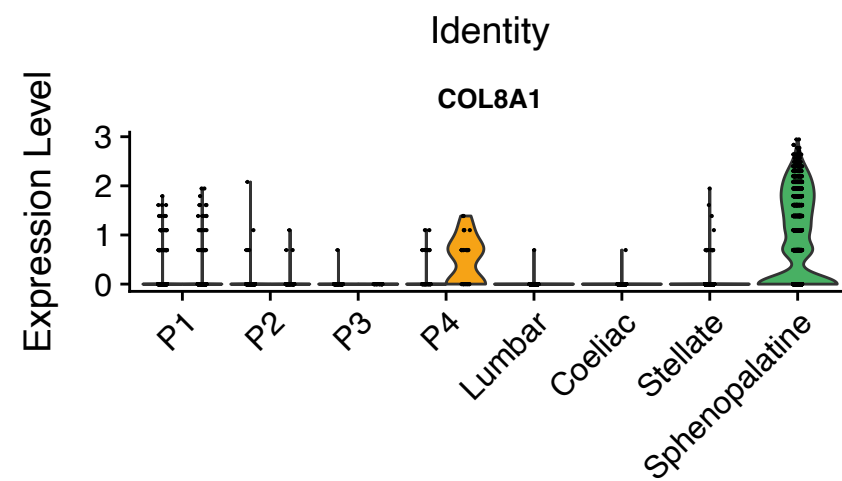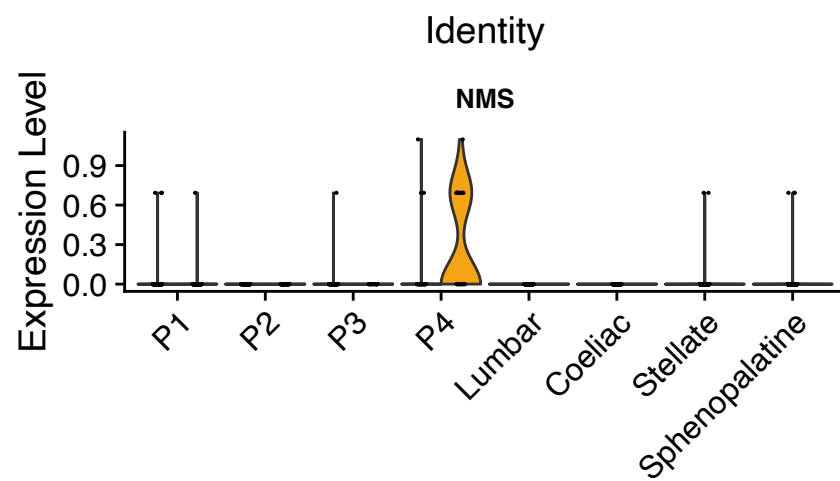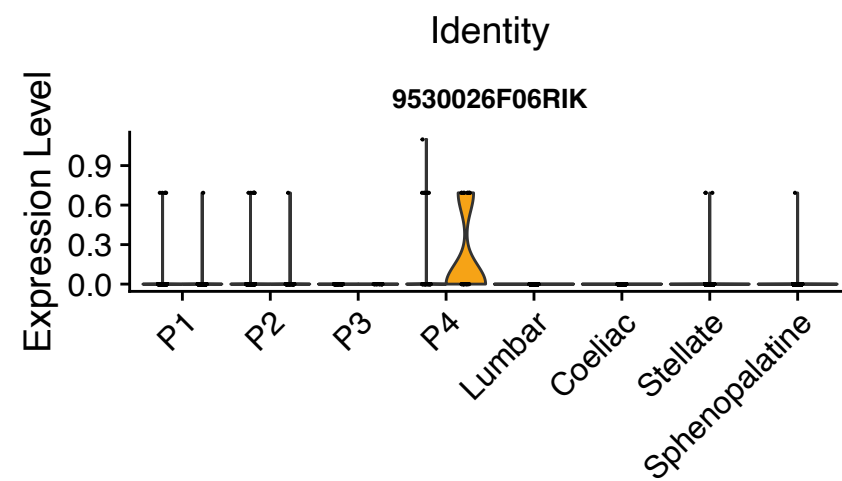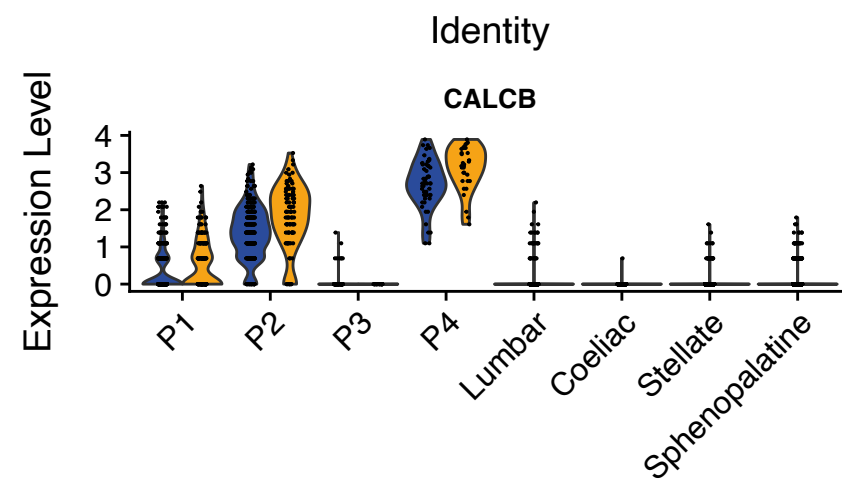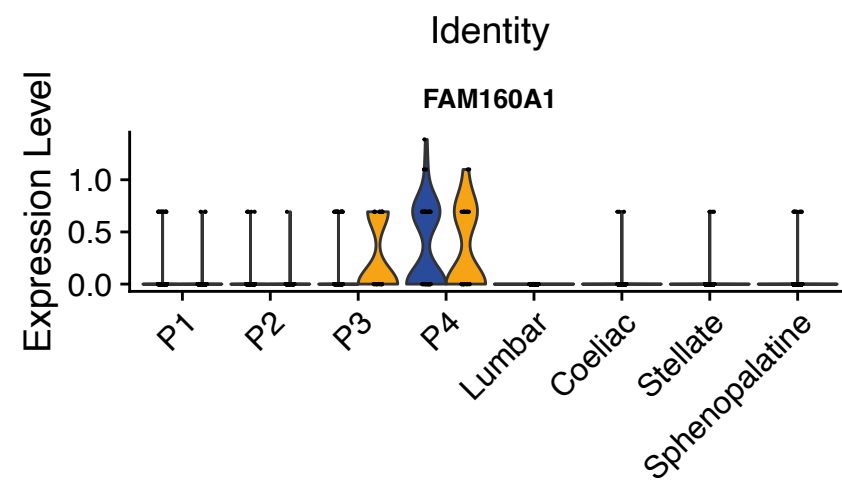

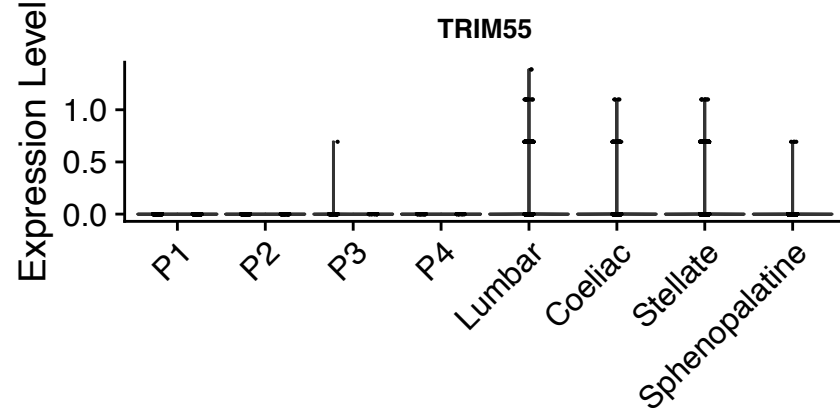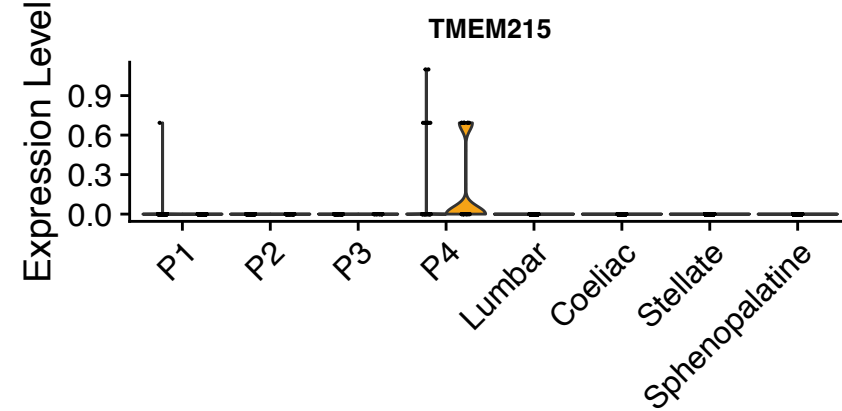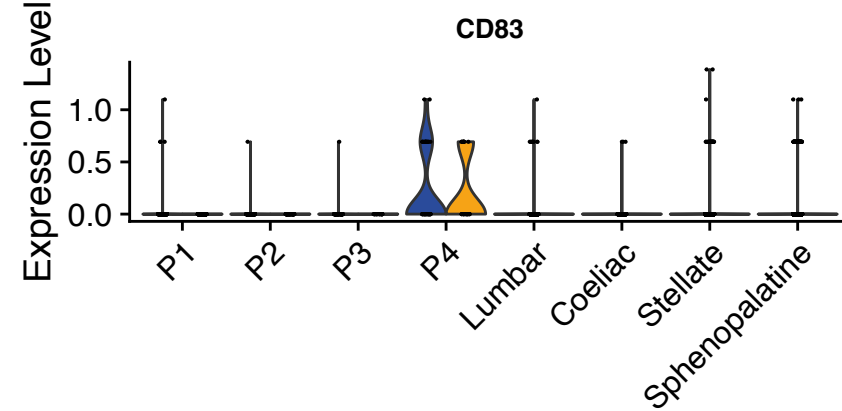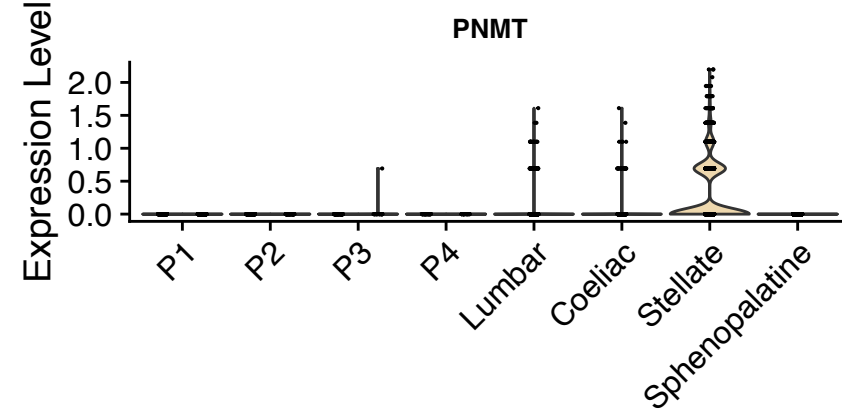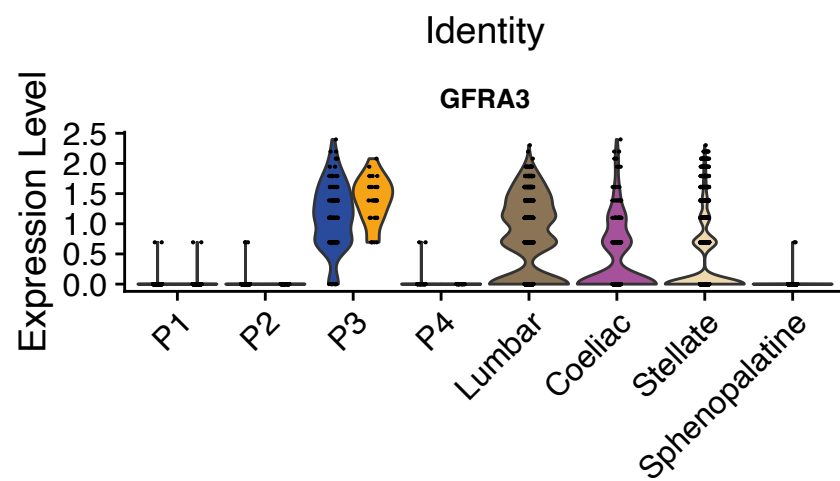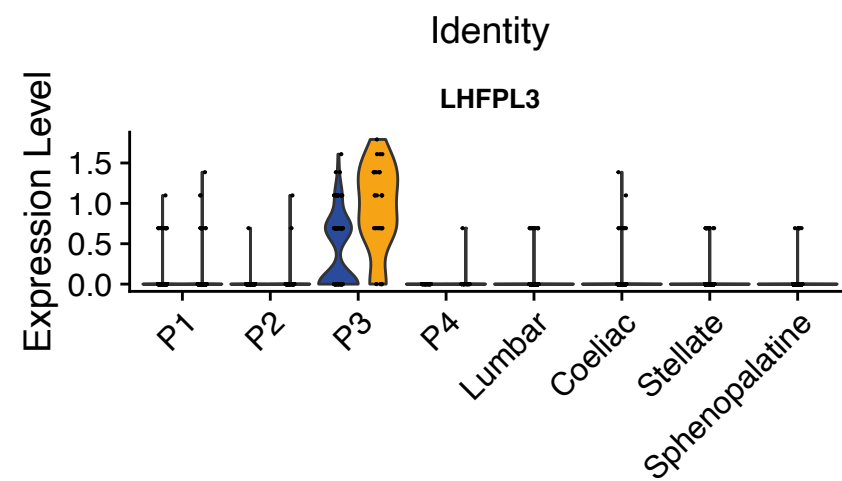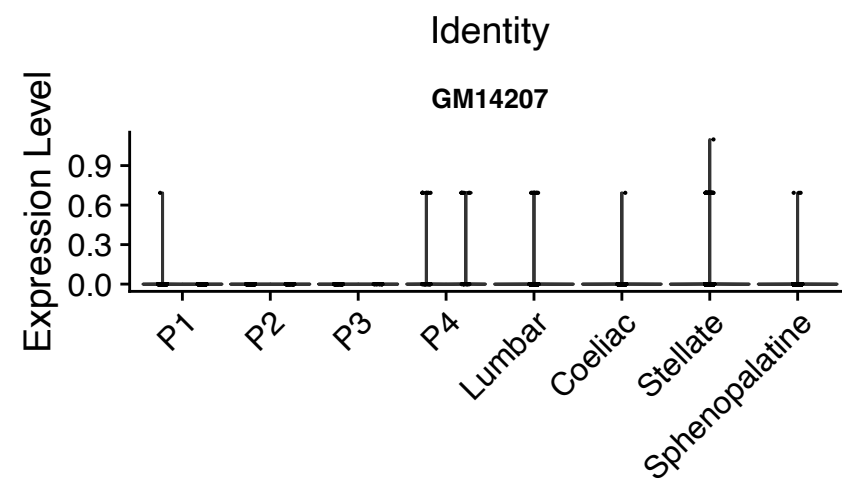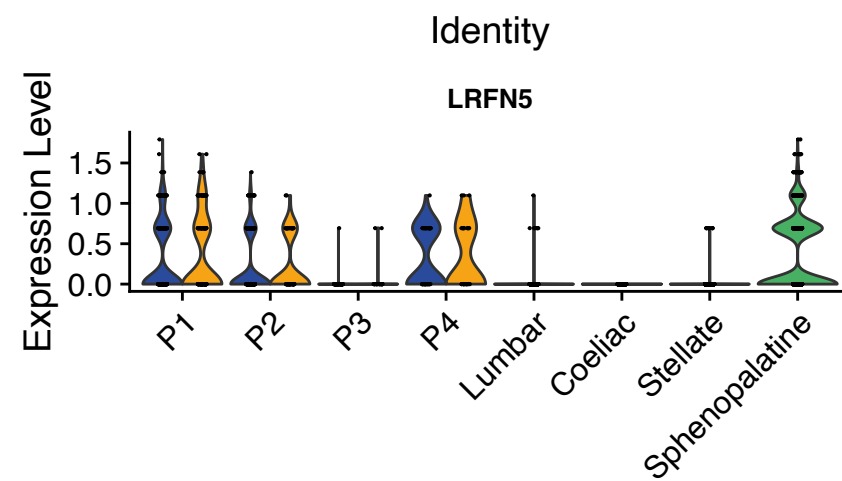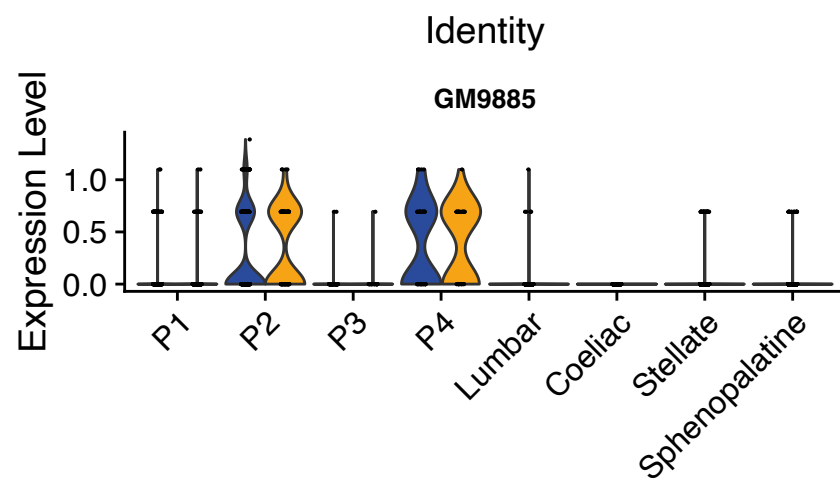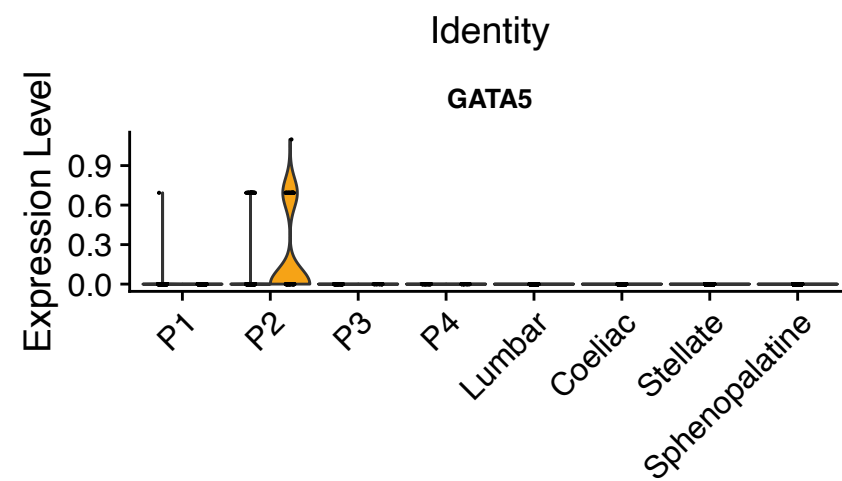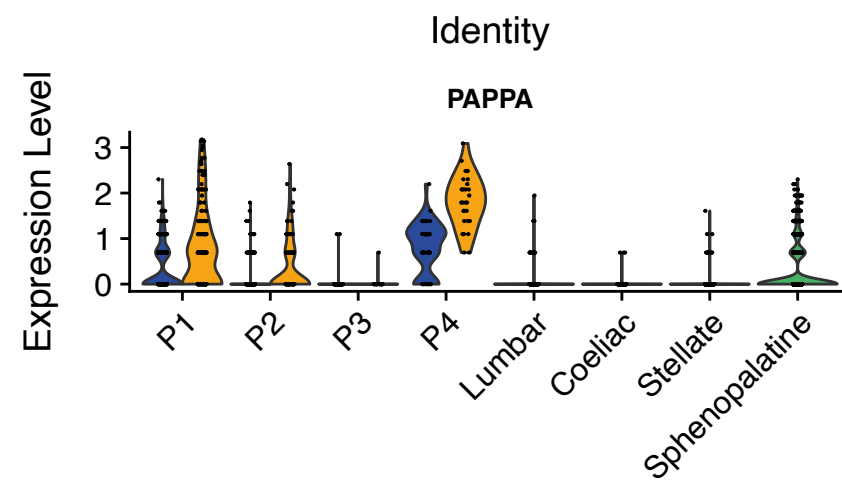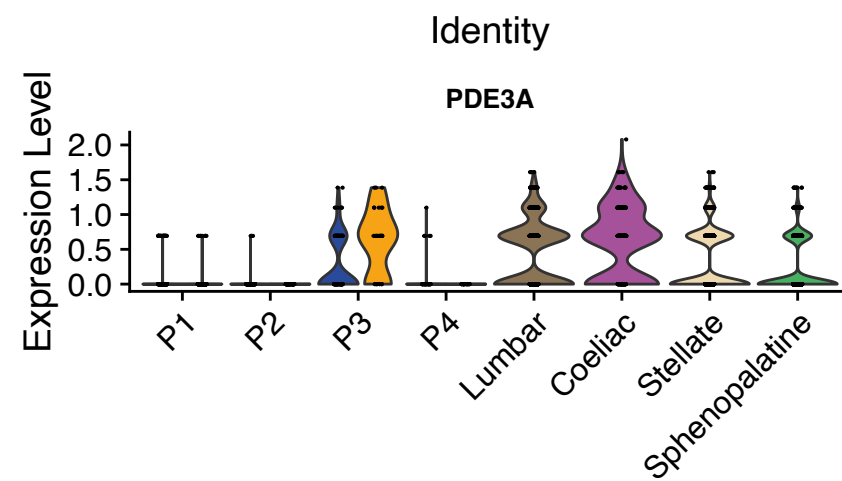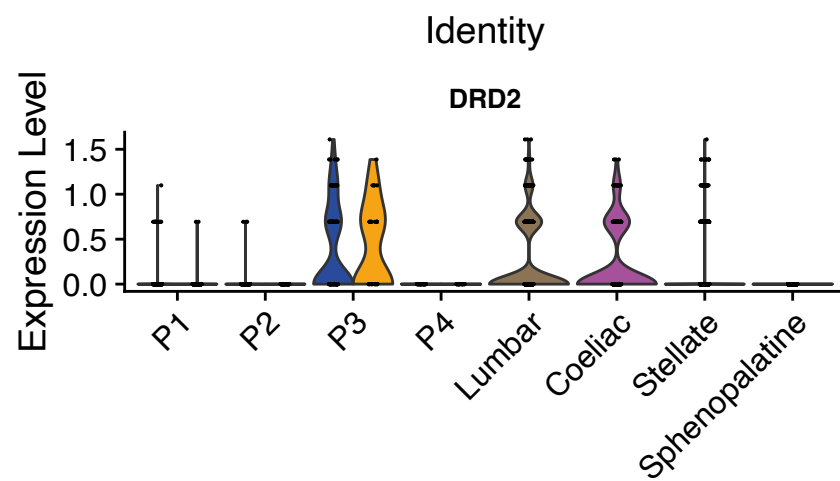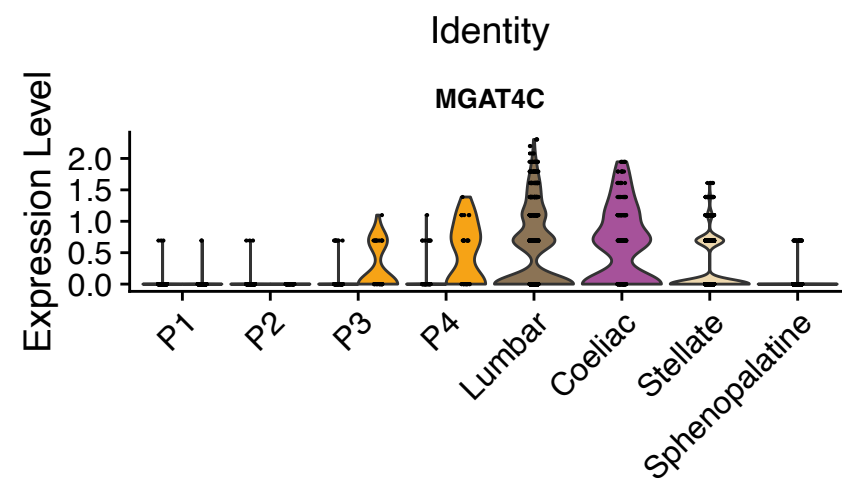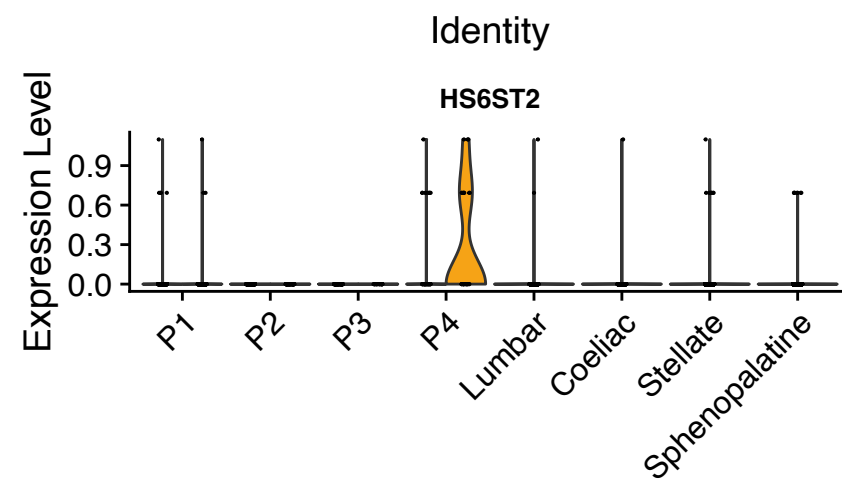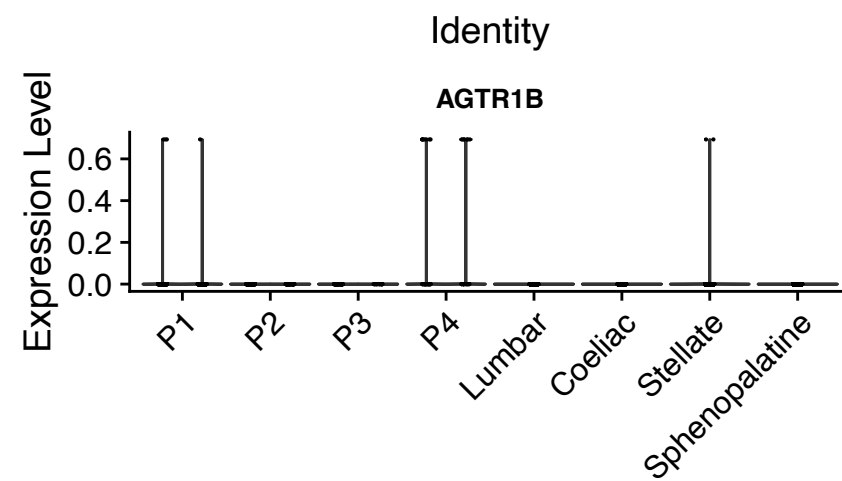

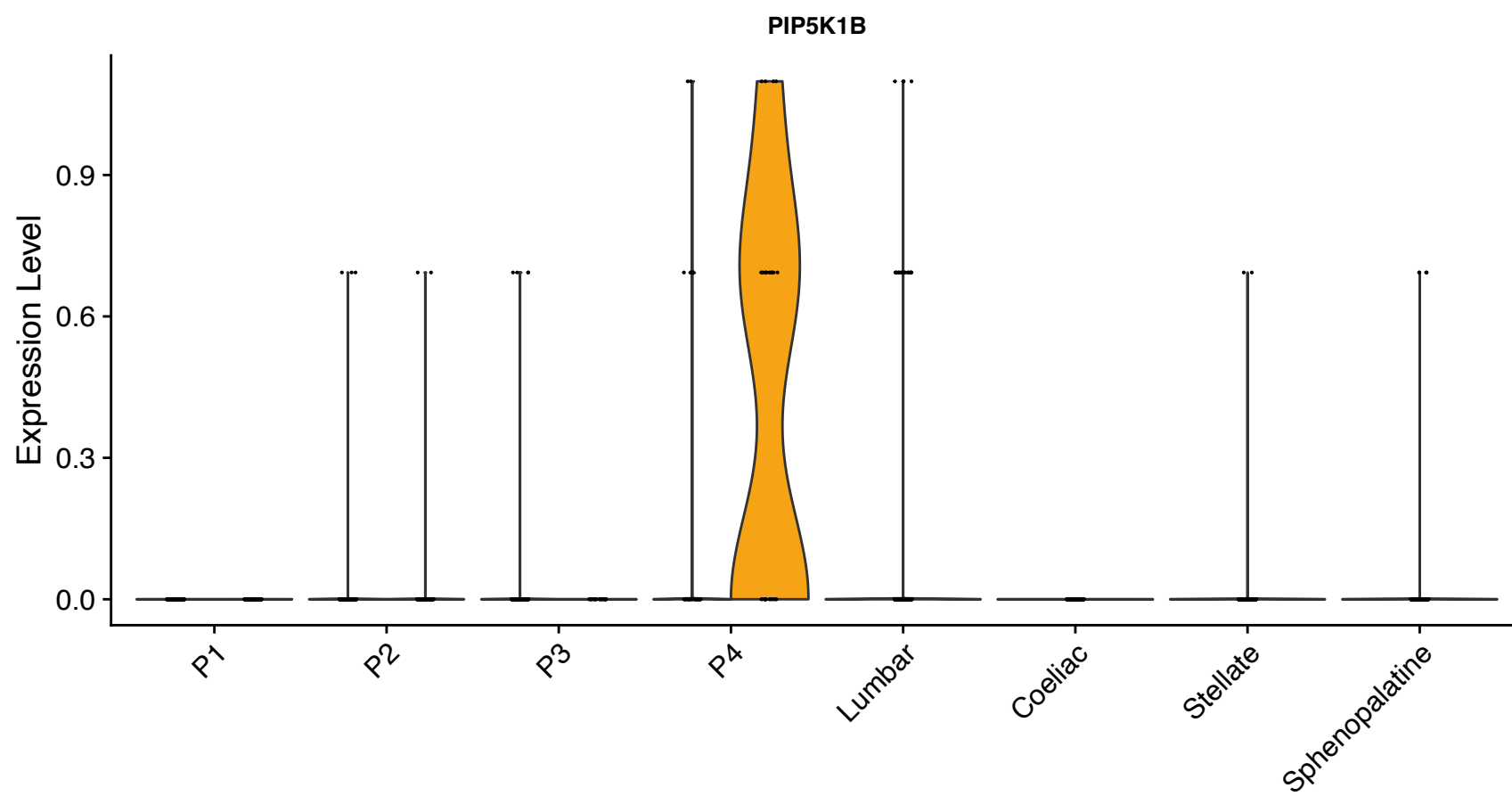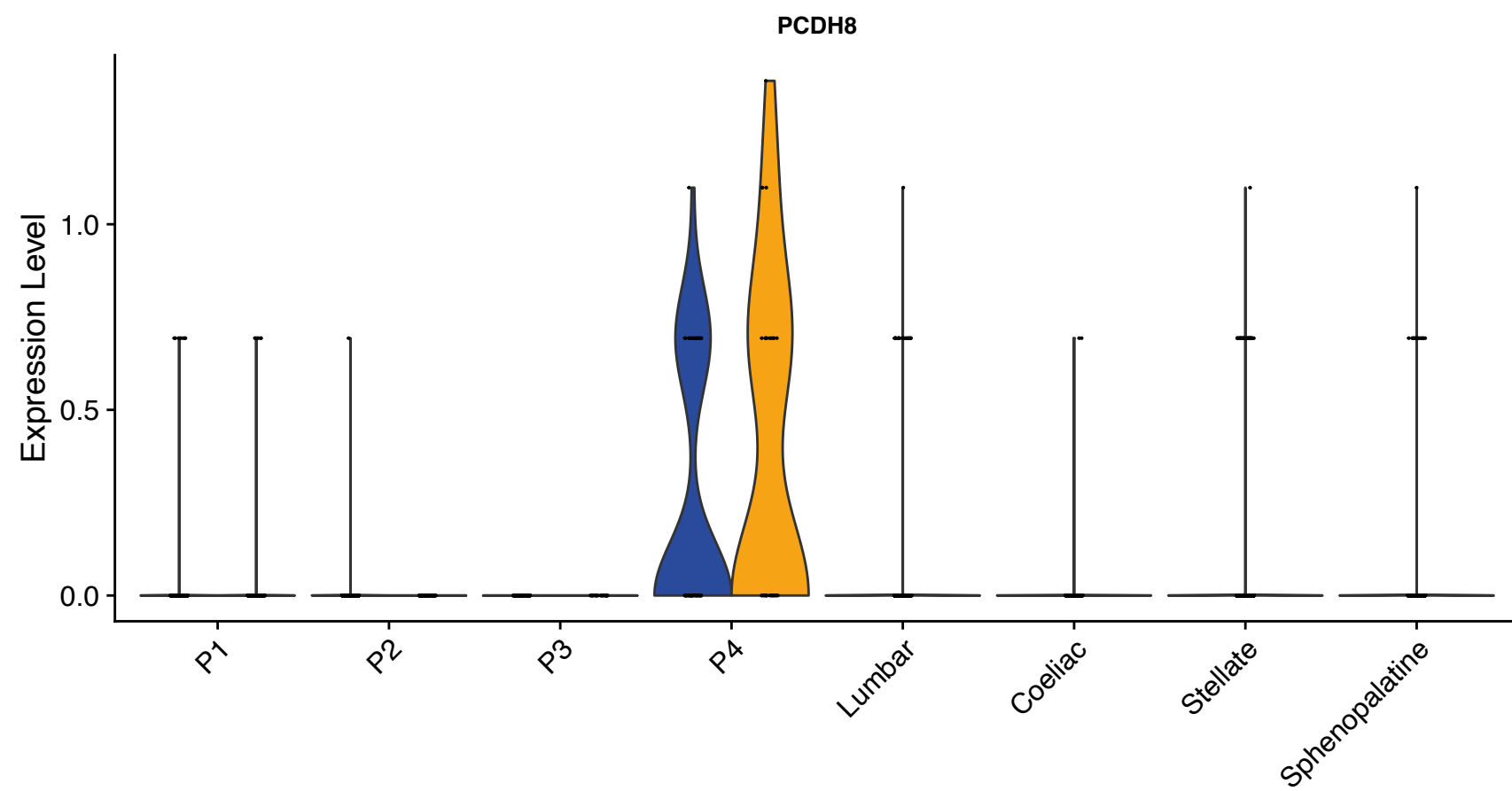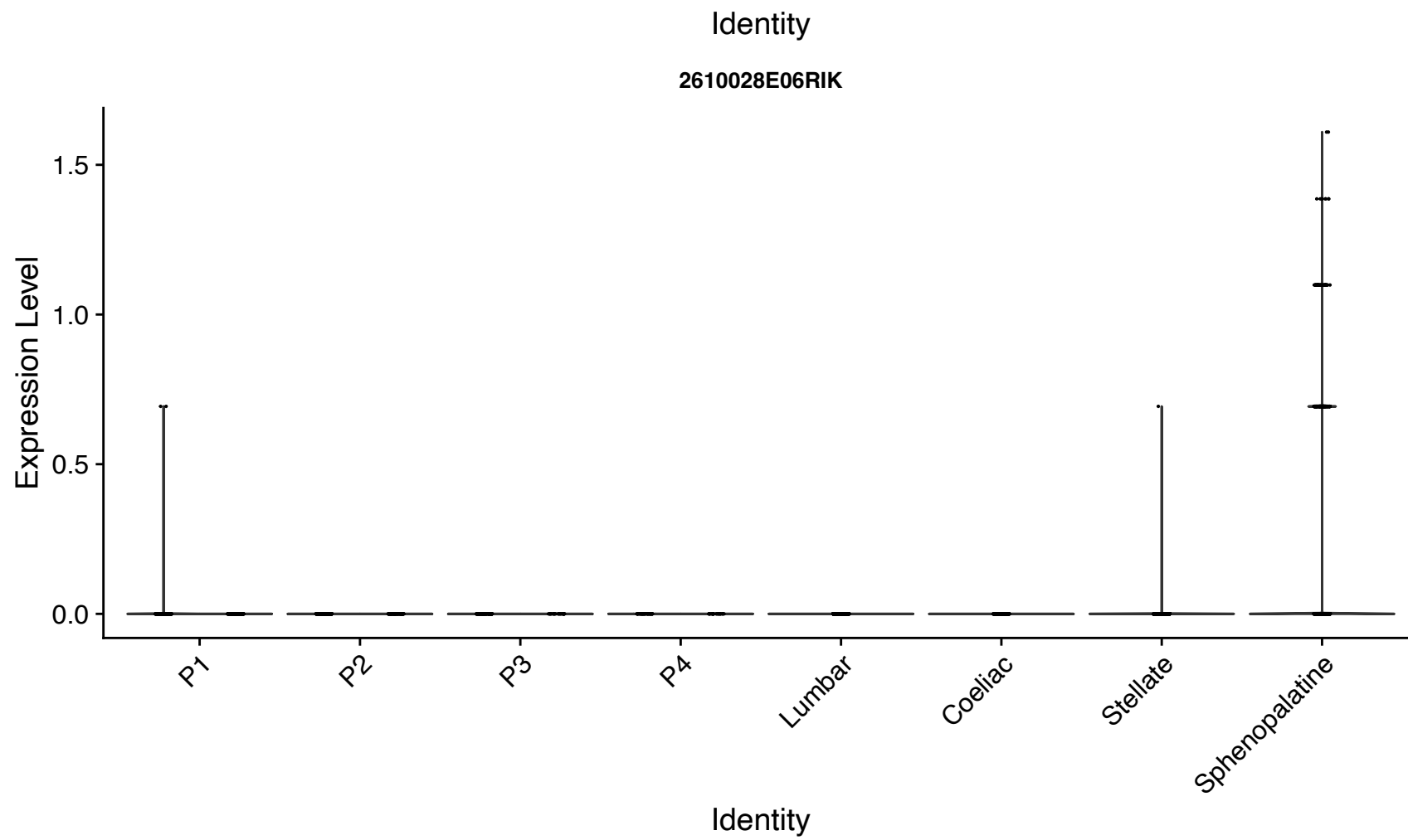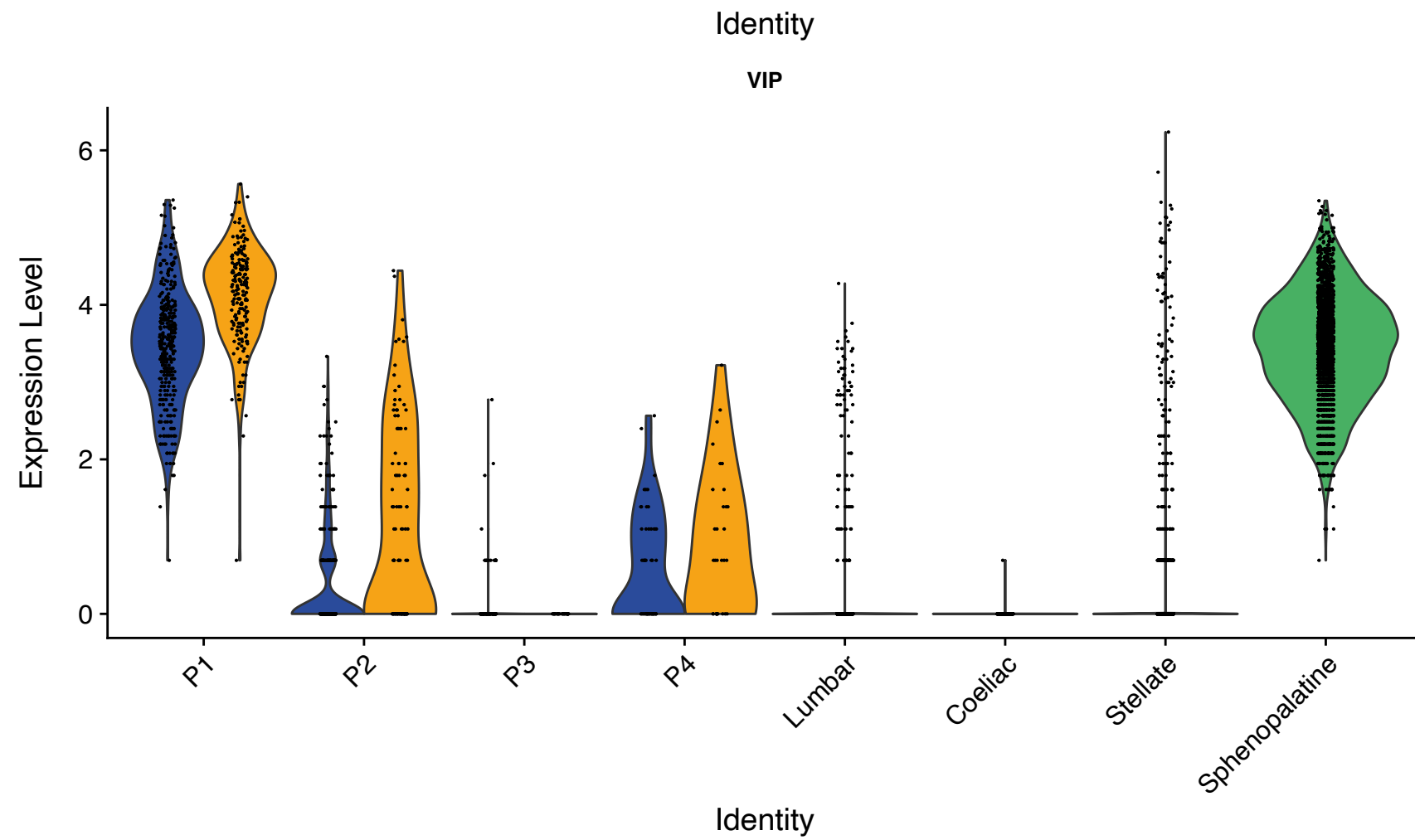

Supplement: Supplementary file 2. [file elife-91576-supp2.pdf]

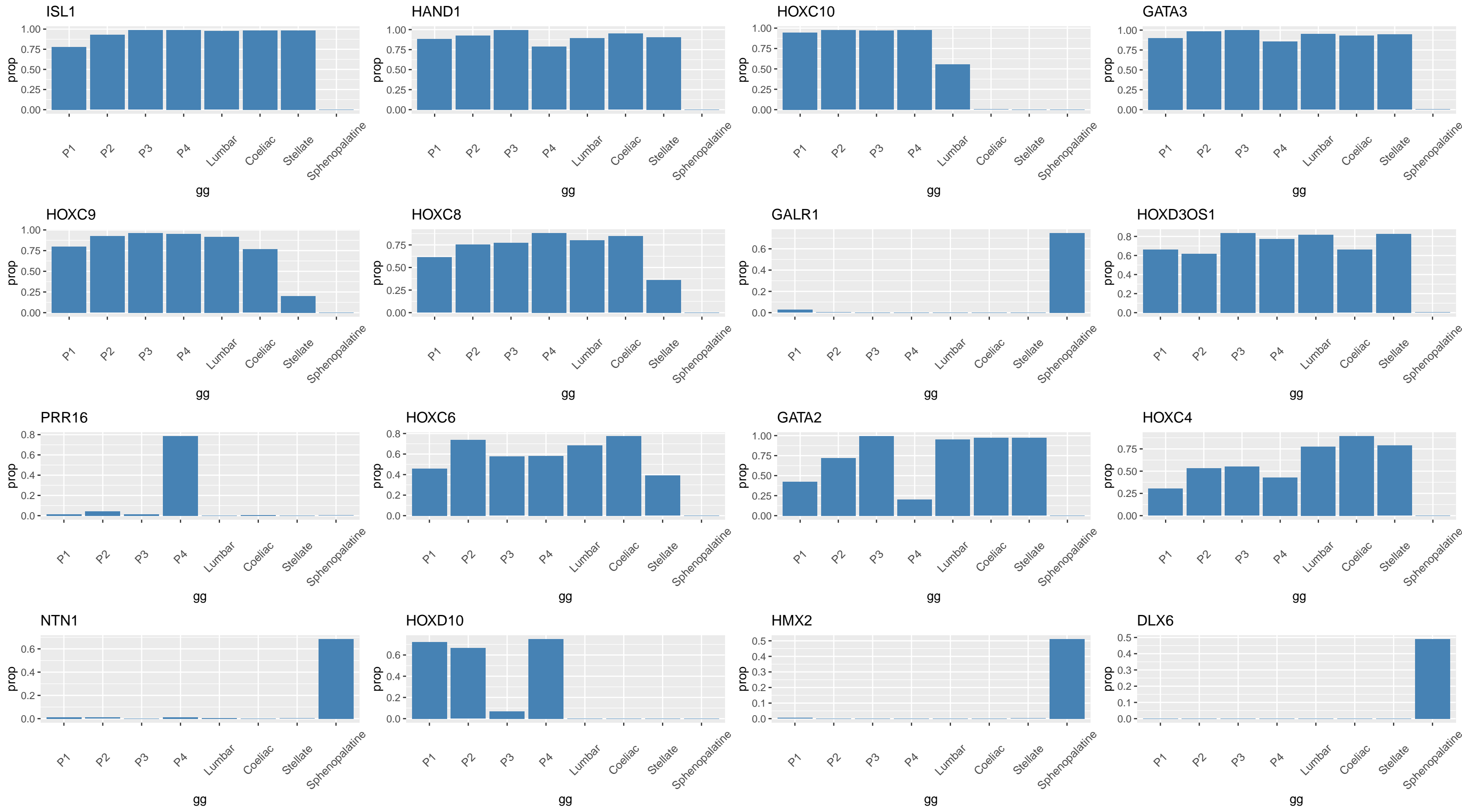

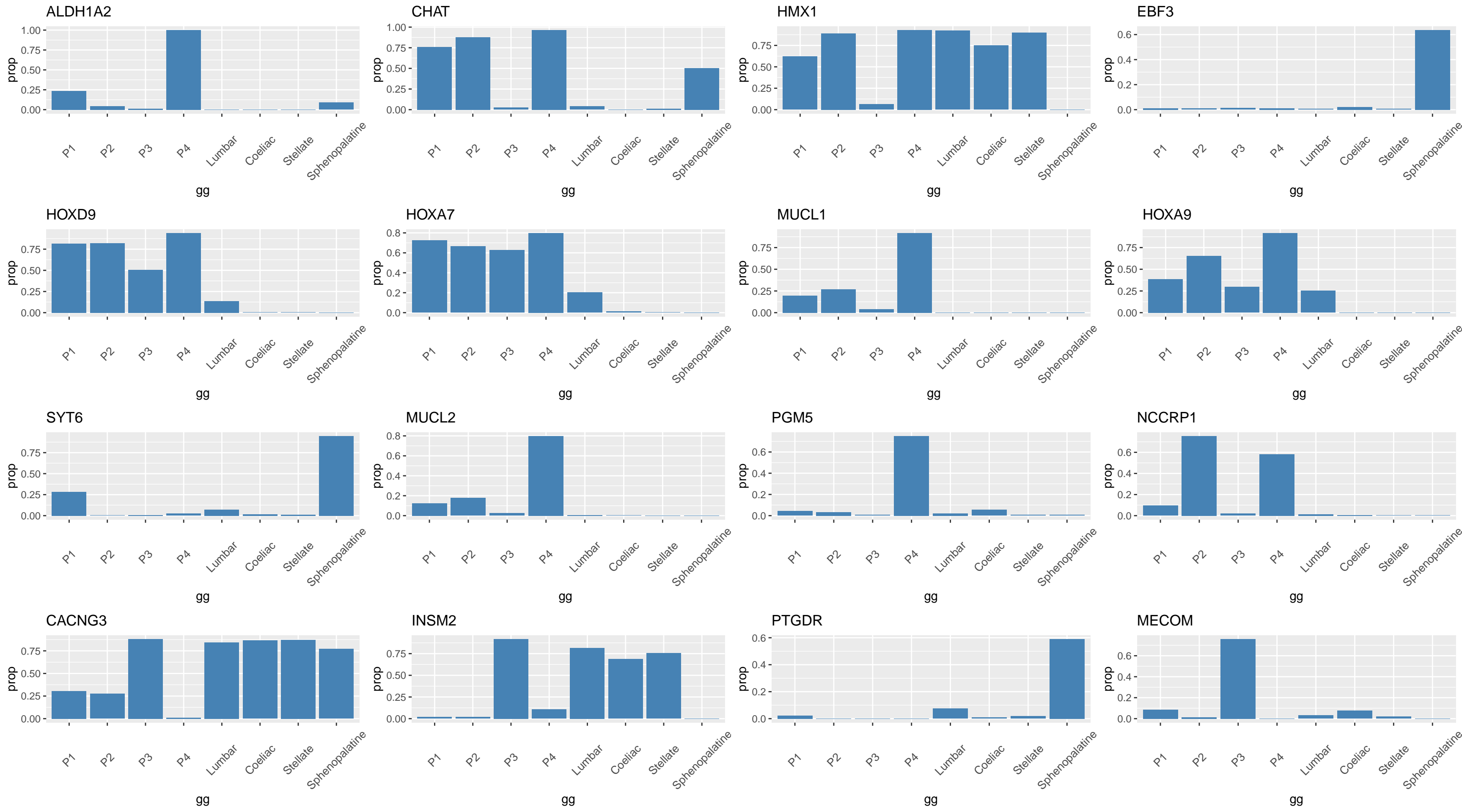

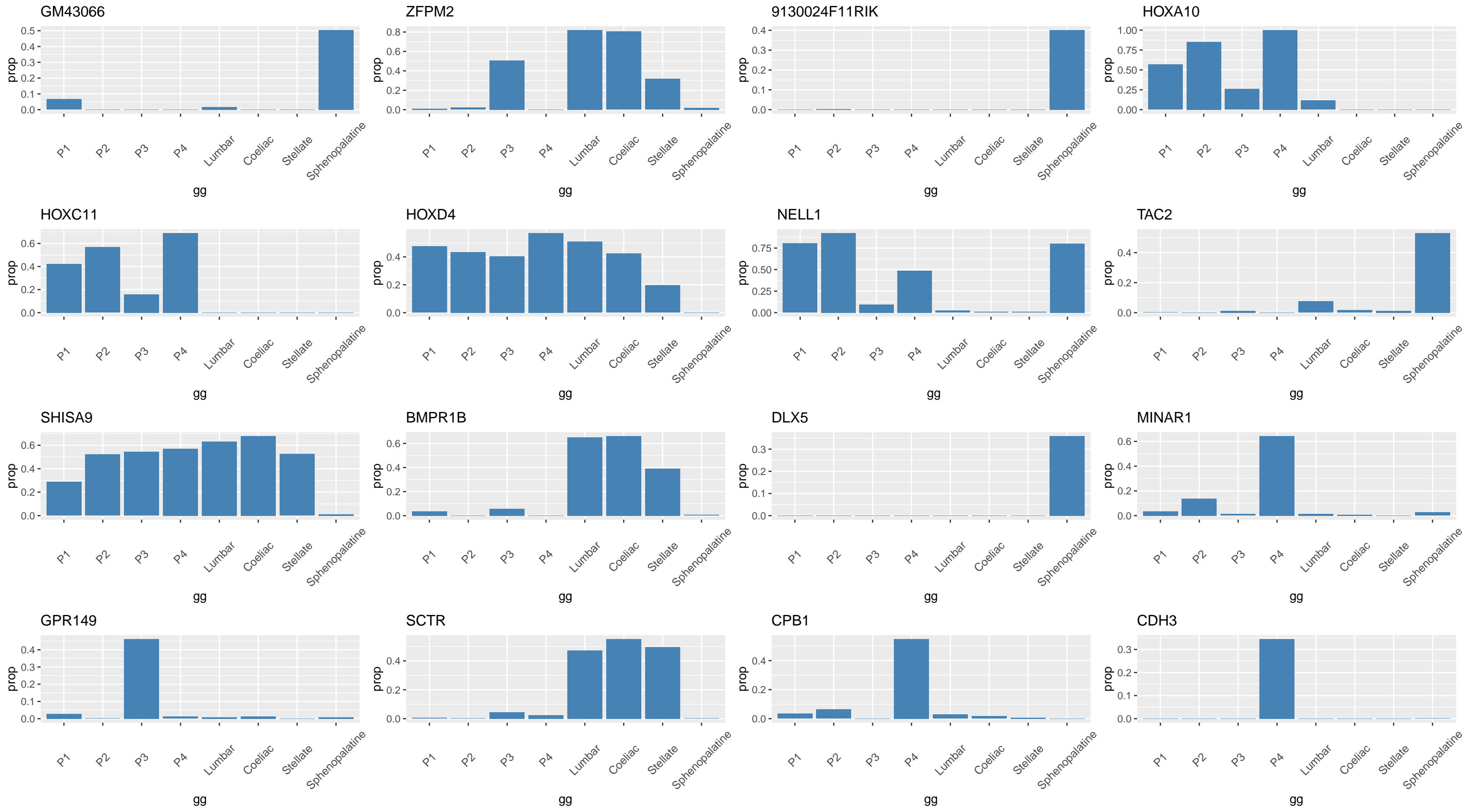

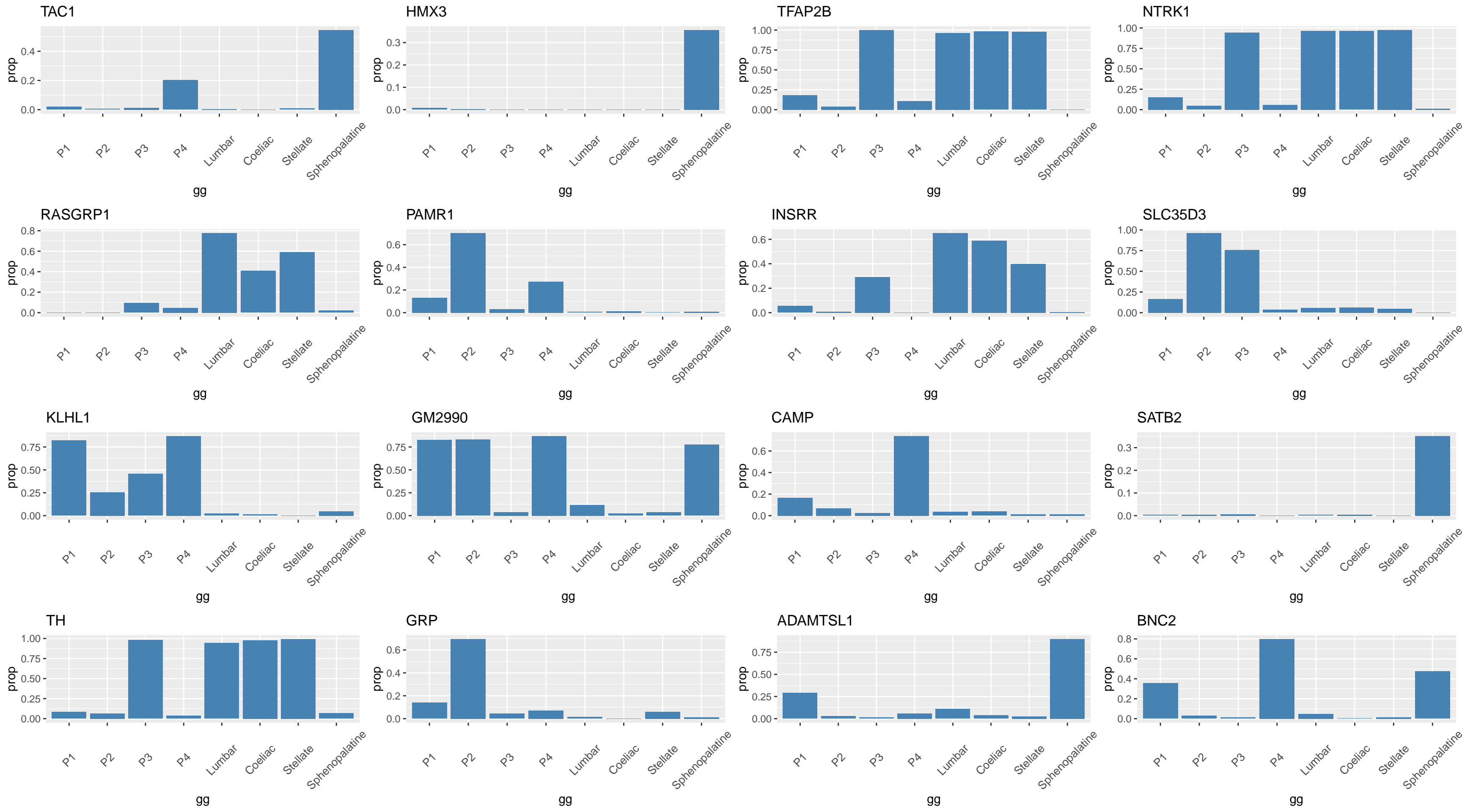

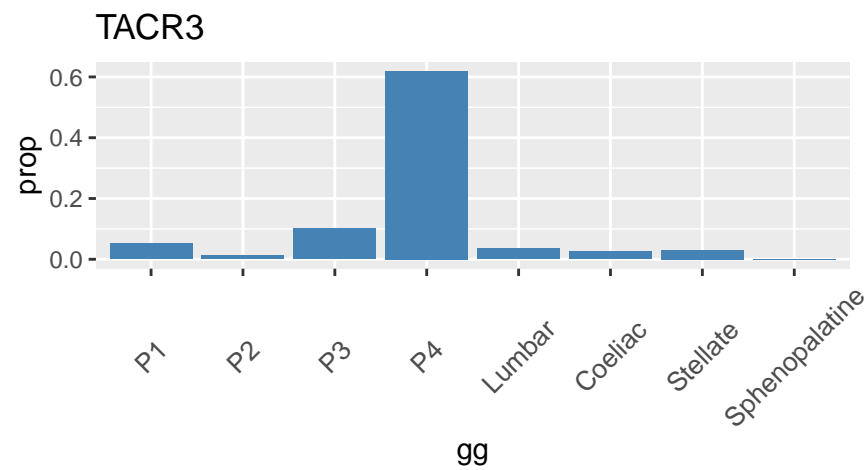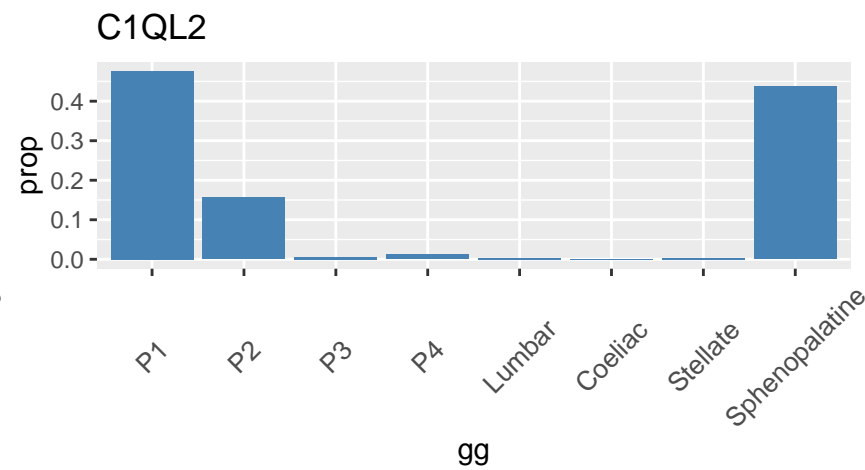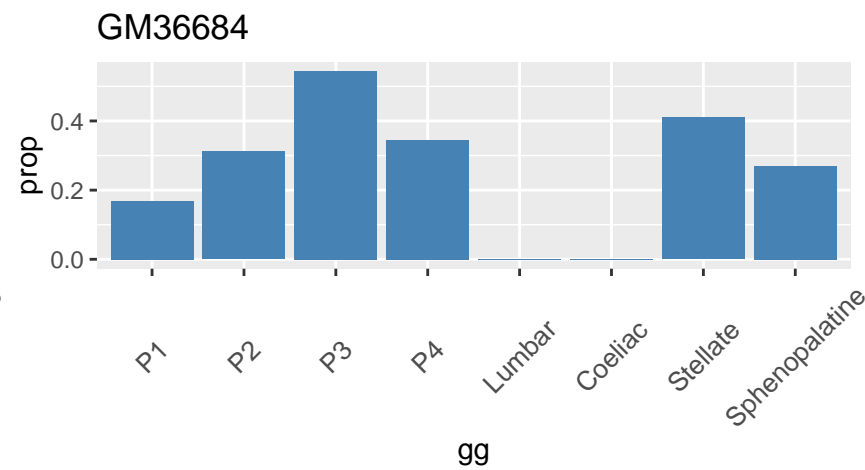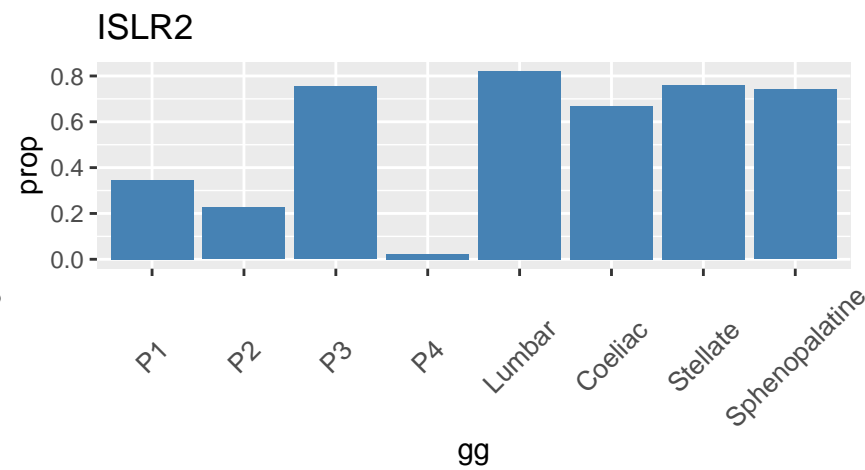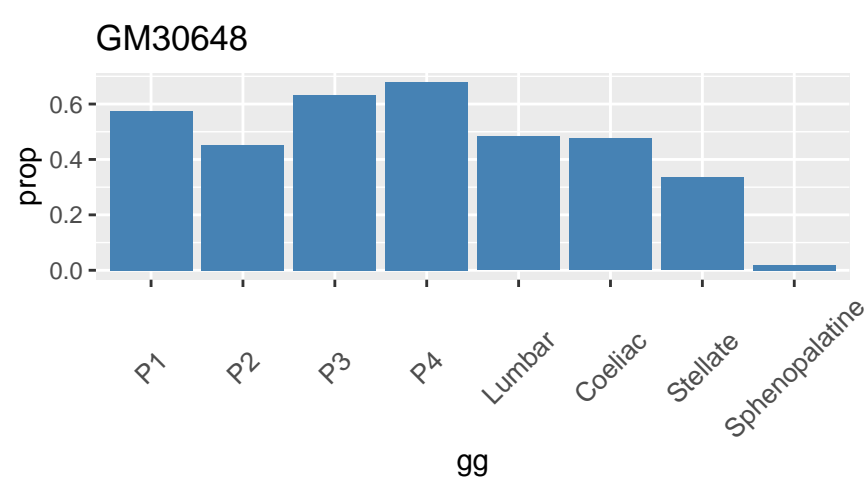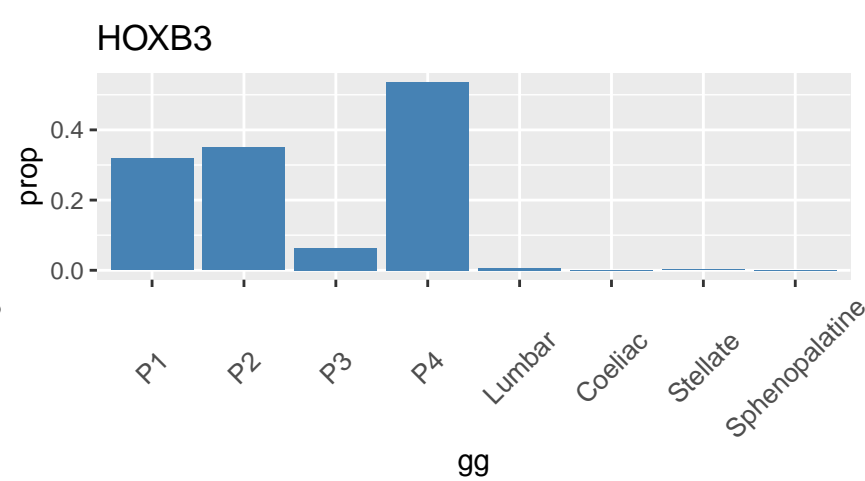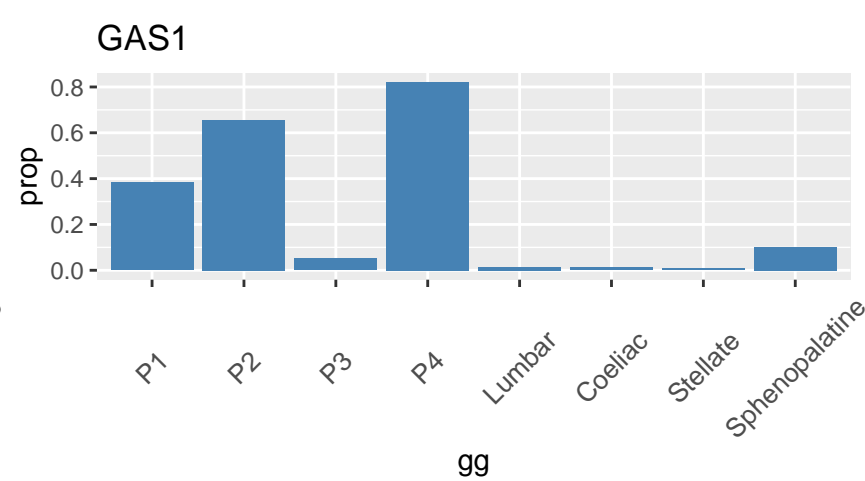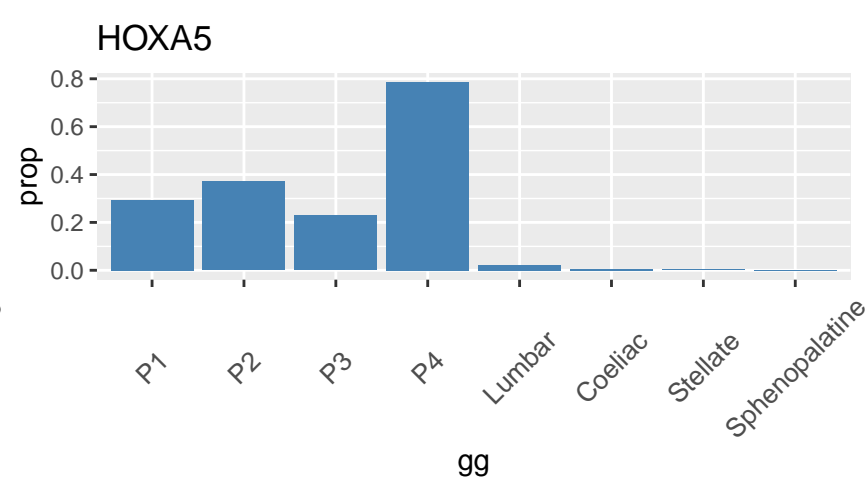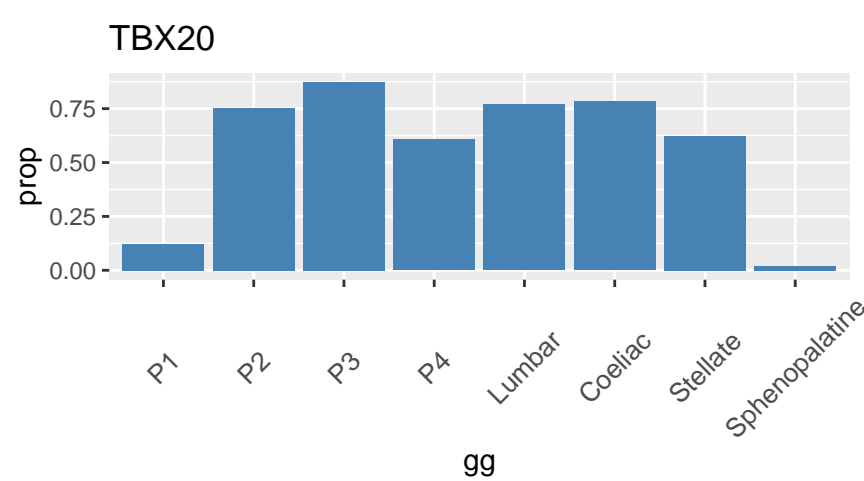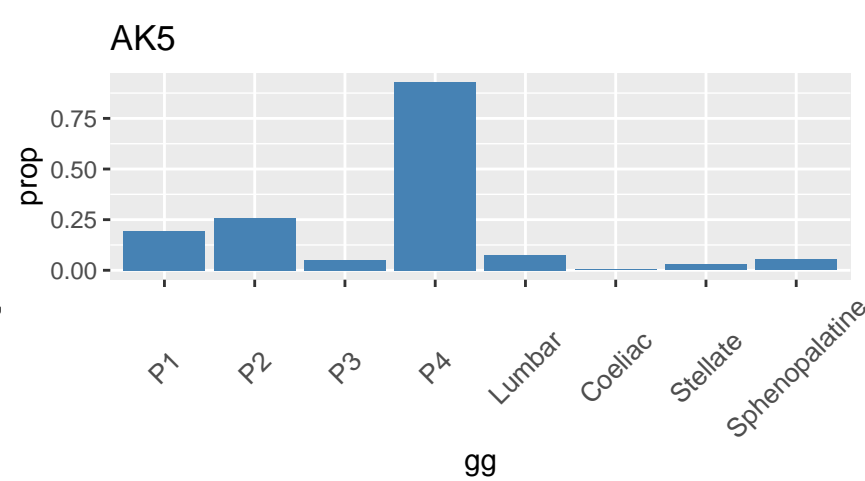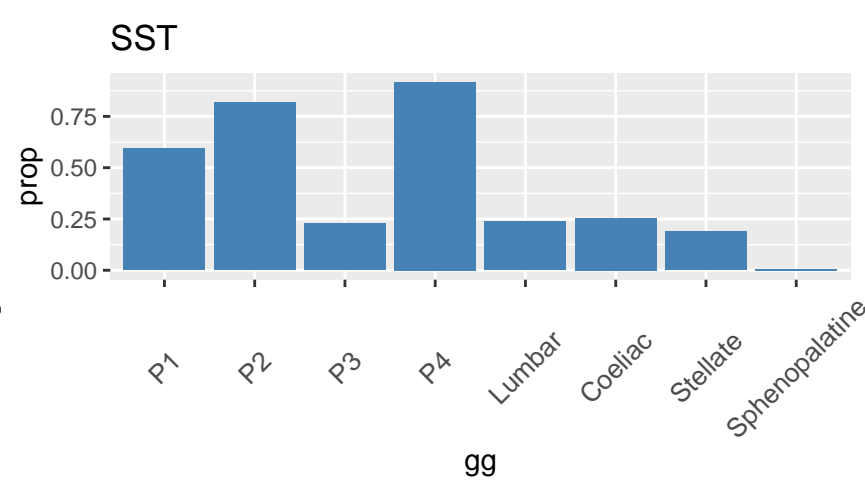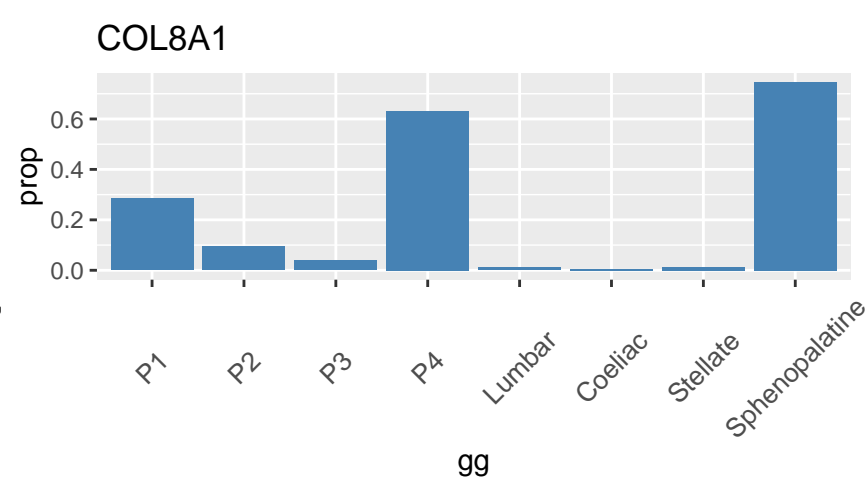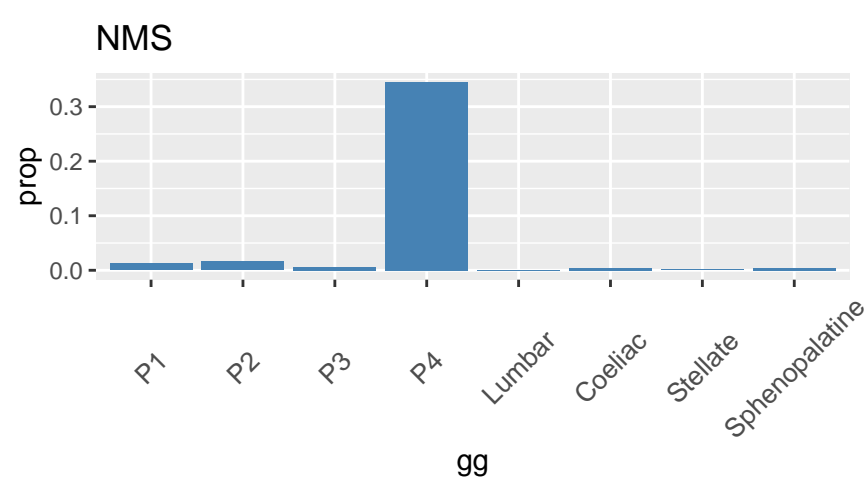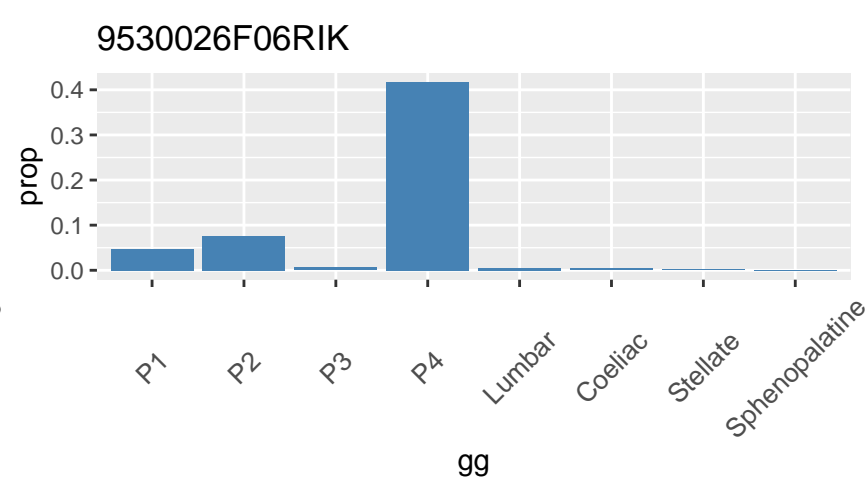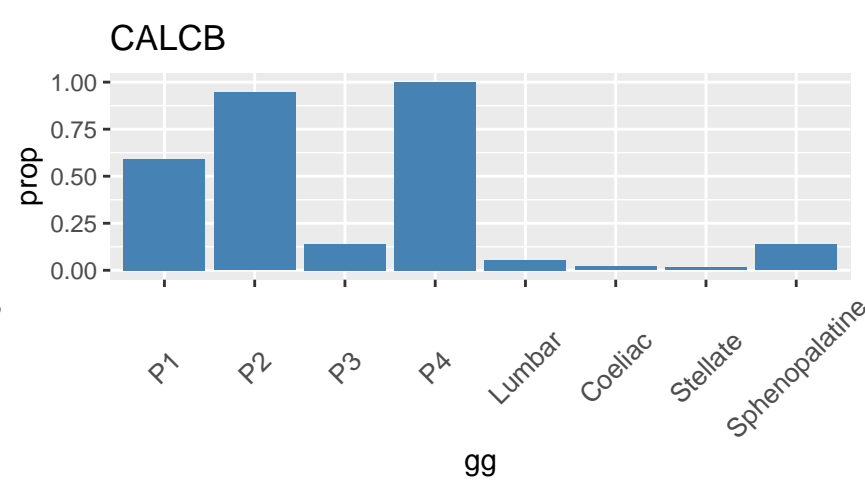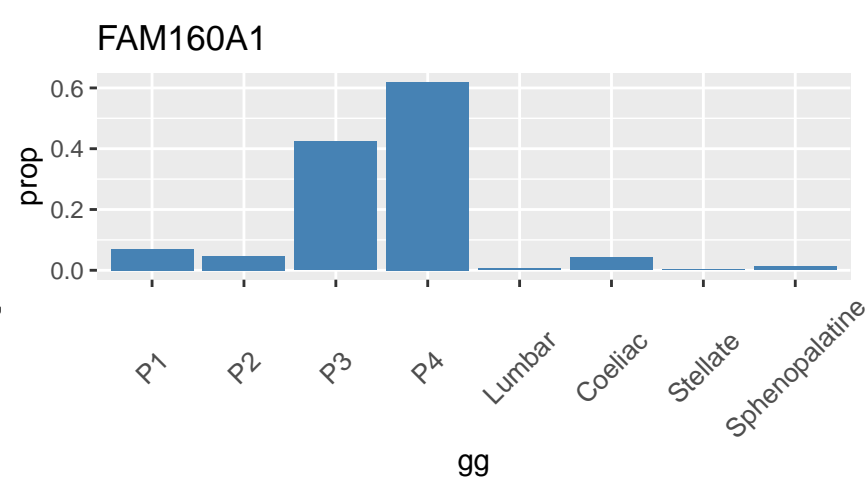

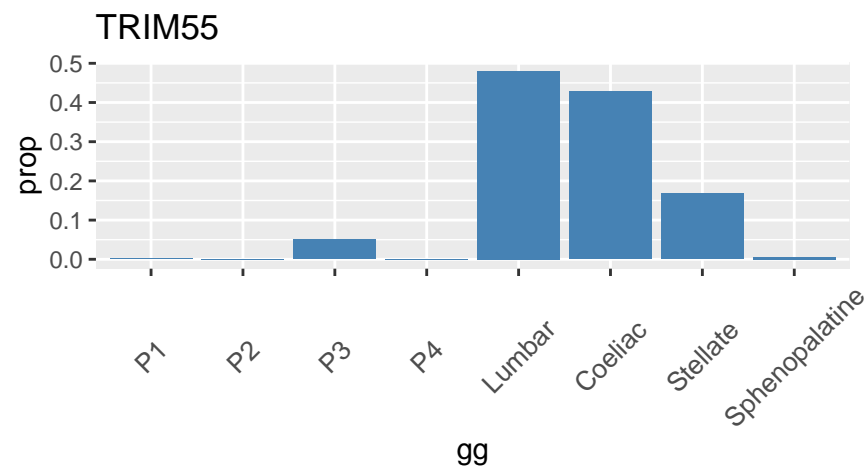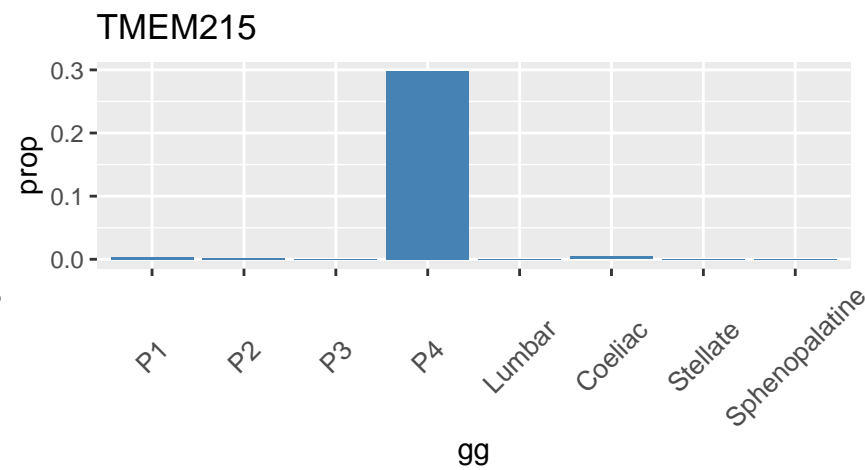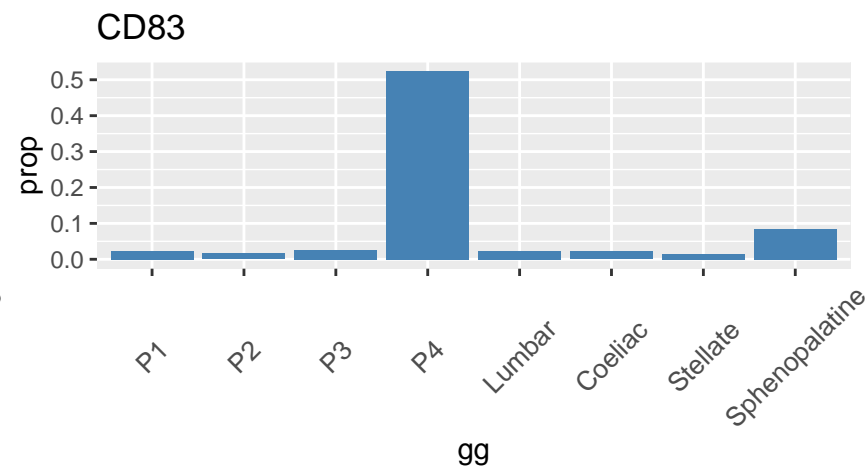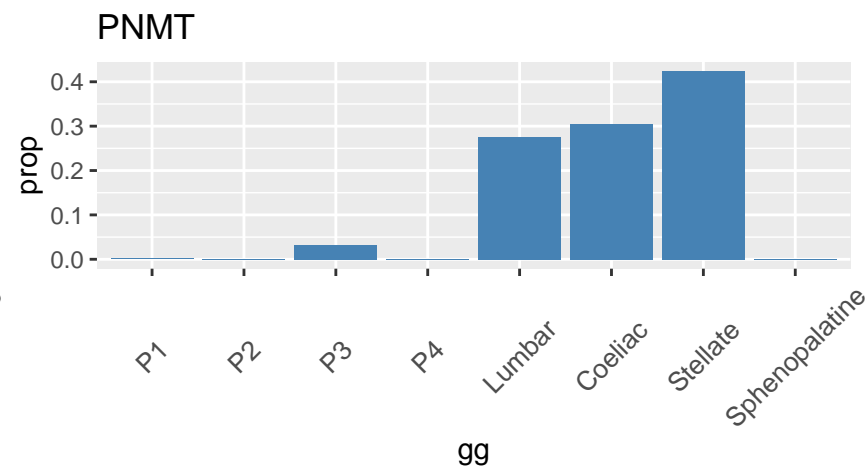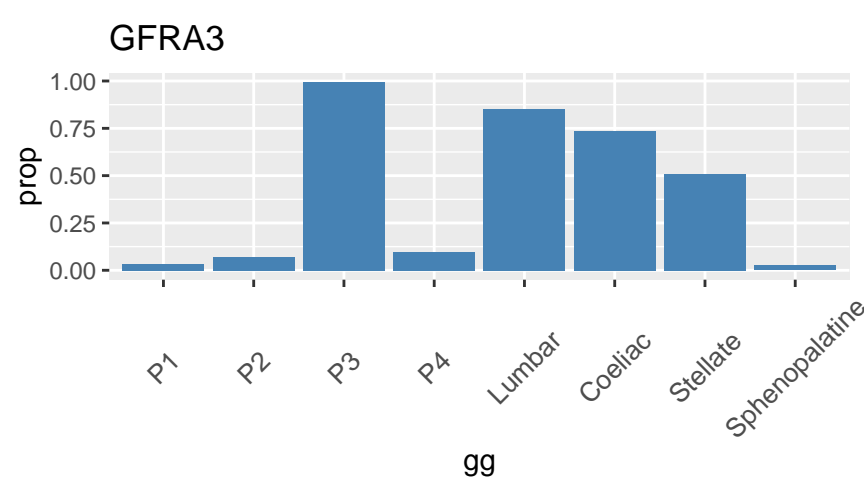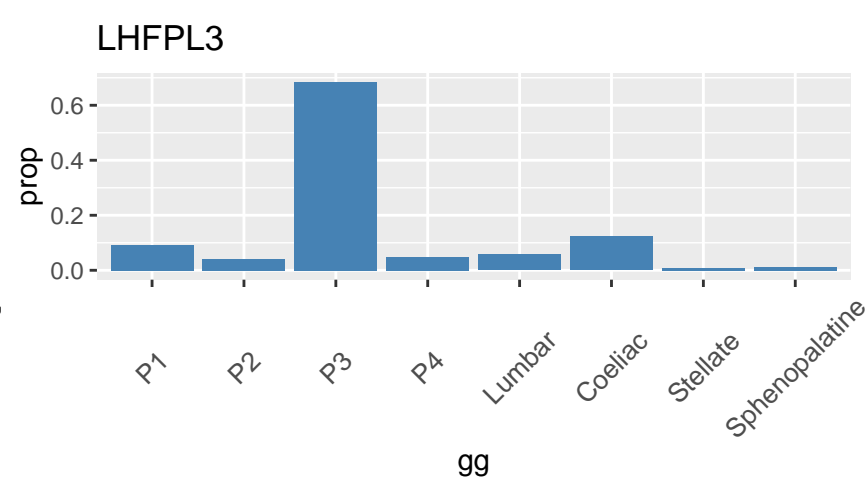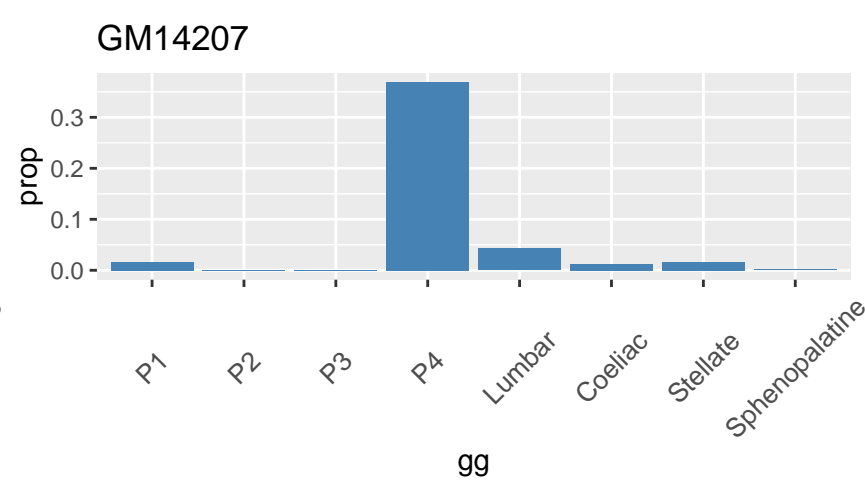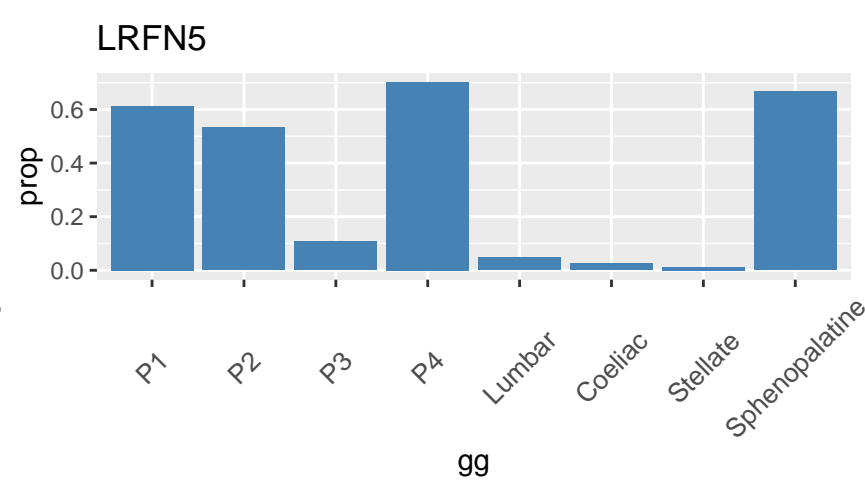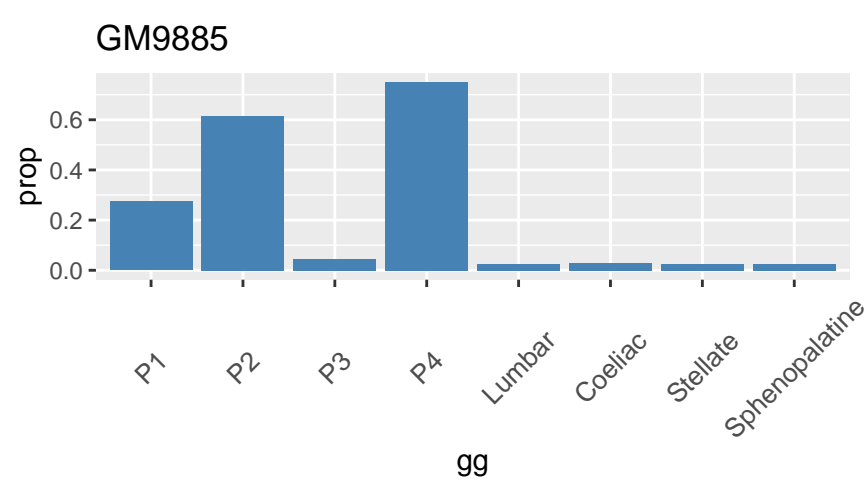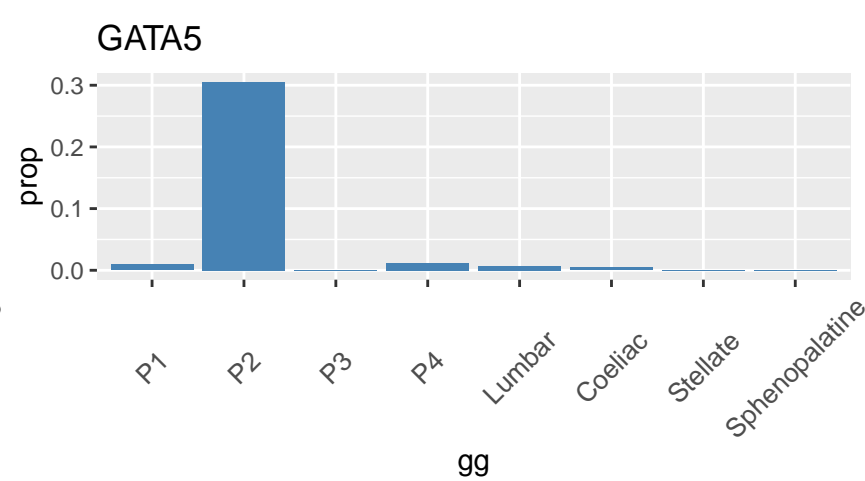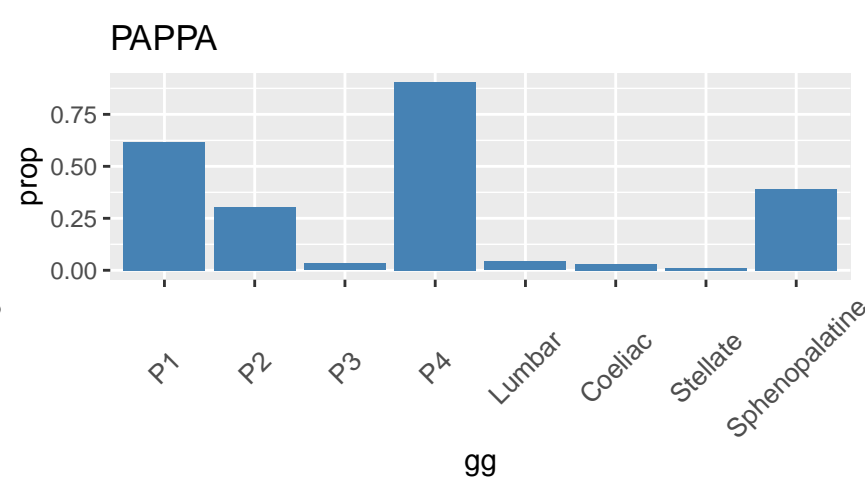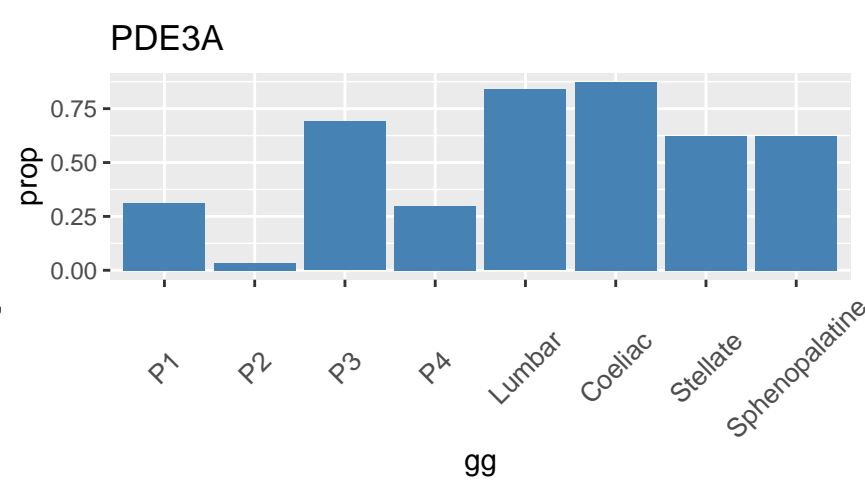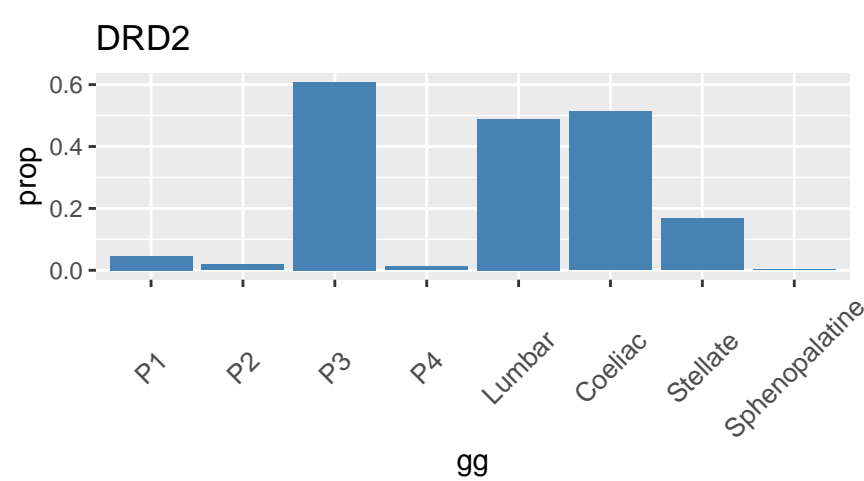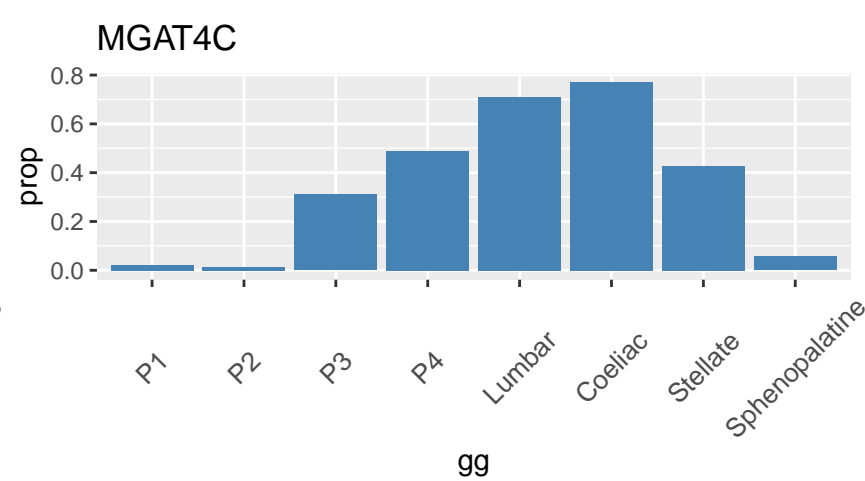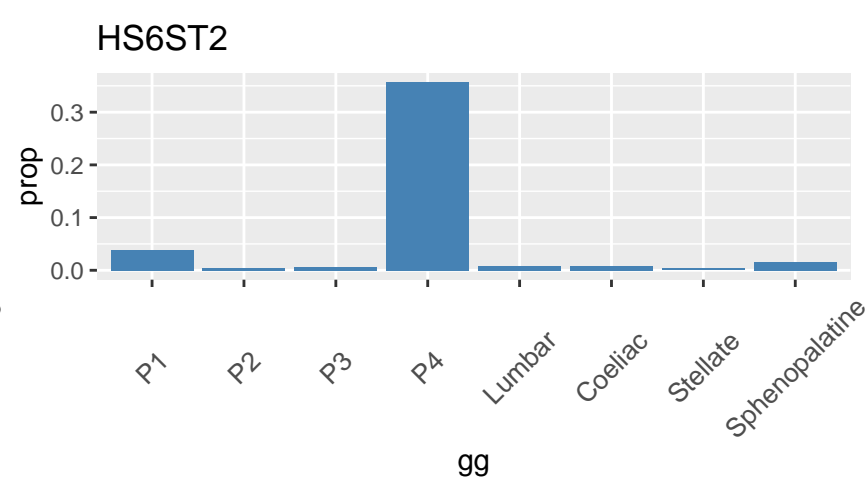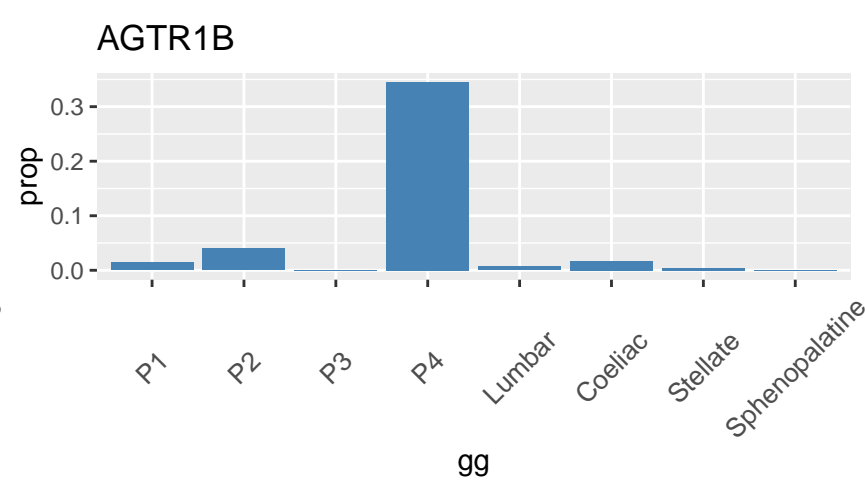

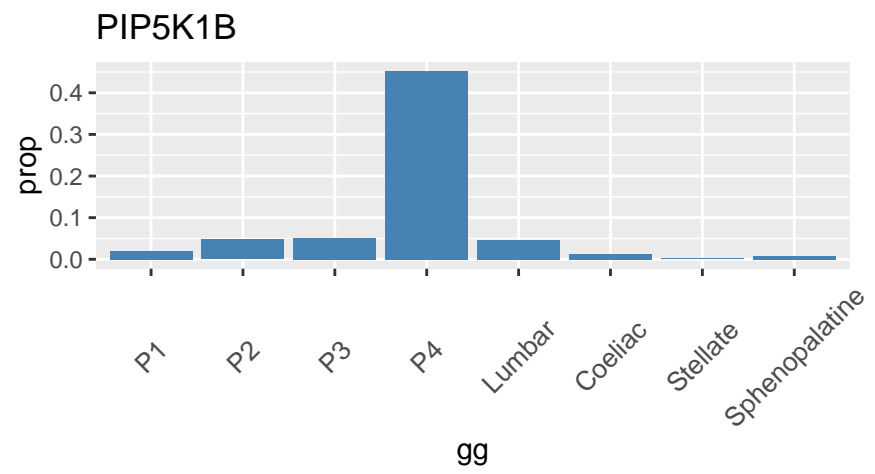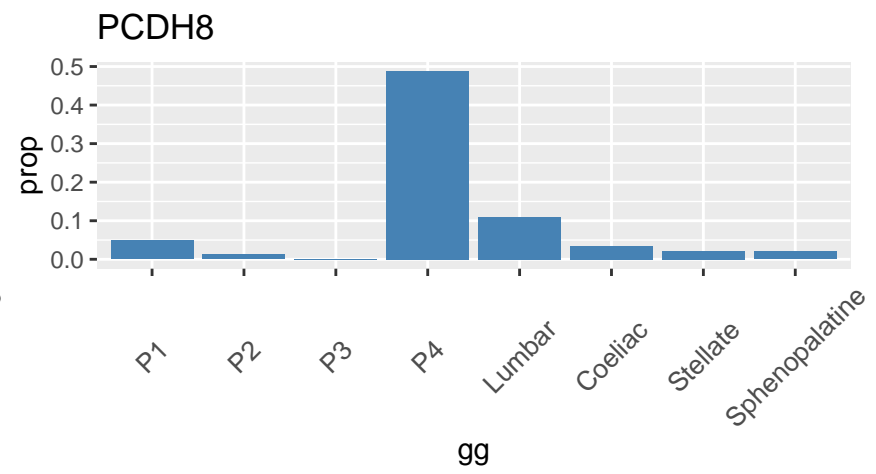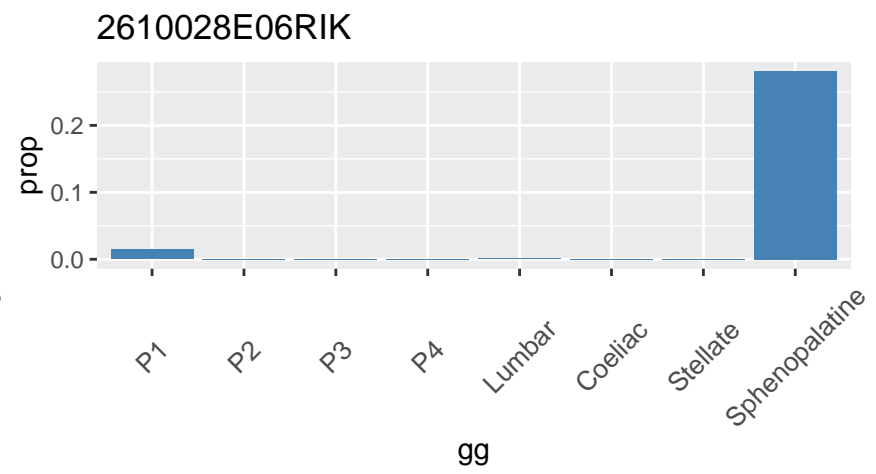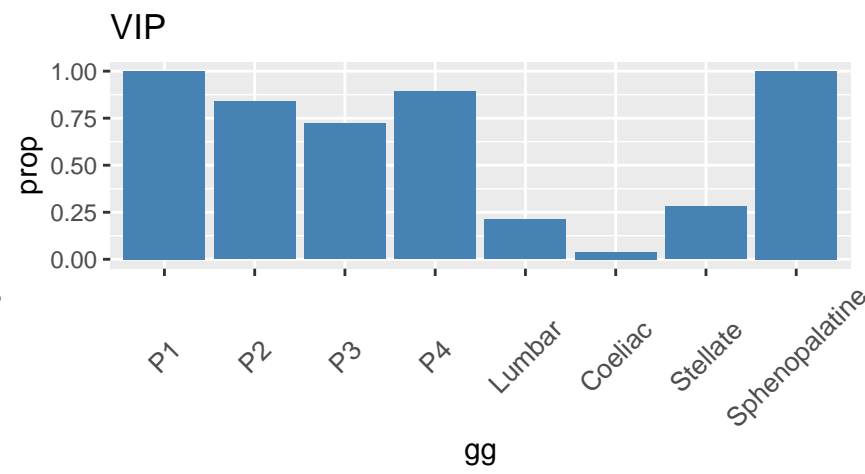

Supplement: Supplementary file 3. [file elife-91576-supp3.pdf]
